# Versatile gold-silver-PB nanojujubes for multi-modal detection and photo-responsive elimination against bacteria

**DOI:** 10.3389/fchem.2023.1211523

**Published:** 2023-05-22

**Authors:** Xining Zhang, Youlin Xiong, Shuangfei Cai, Ting Wu, Zheng Lian, Chen Wang, Wei Zhang, Rong Yang

**Affiliations:** ^1^ CAS Key Laboratory for Biomedical Effects of Nanomaterials and Nanosafety, Center of Materials Science and Optoelectronics Engineering, CAS Center for Excellence in Nanoscience, National Center for Nanoscience and Technology, University of Chinese Academy of Sciences, Beijing, China; ^2^ Sino-Danish College, Sino-Danish Center for Education and Research, University of Chinese Academy of Sciences, Beijing, China; ^3^ Institute of Applied Physics and Computational Mathematics, Beijing, China

**Keywords:** gold nanobipyramids, prussian blue, nanozyme, SERS, bacterial detection, sterilization

## Abstract

Bacterial infections have become a serious threat to global public health. Nanomaterials have shown promise in the development of bacterial biosensing and antibiotic-free antibacterial modalities, but single-component materials are often less functional and difficult to achieve dual bacterial detection and killing. Herein, we report a novel strategy based on the effective integration of multi-modal bacterial detection and elimination, by constructing the versatile gold-silver-Prussian blue nanojujubes (GSP NJs) via a facile template etching method. Such incorporation of multi-components involves the utilization of cores of gold nanobipyramids with strong surface-enhanced Raman scattering (SERS) activity, the shells of Prussian blue as both an efficient bio-silent SERS label and an active peroxidase-mimic, and functionalization of polyvinyl pyrrolidone and vancomycin, respectively endowing them with good colloidal dispersibility and specificity against *S. aureus*. The GSP NJs show operational convenience in the SERS detection and excellent peroxidase-like activity for the sensitive colorimetric detection. Meanwhile, they exhibit robust near-infrared photothermal/photodynamic effects, and the photo-promoted Ag^+^ ions release, ultimately achieving a high antibacterial efficiency over 99.9% in 5 min. The NJs can also effectively eliminate complex biofilms. The work provides new insights into the design of multifunctional core-shell nanostructures for the integrated bacterial detection and therapy.

## 1 Introduction

Bacterial infections have become a great threat to global public health, owing to the challenges posed by antimicrobial resistance to the conventional antibiotics (Doolan et al., 2022); accordingly, new and efficient strategies for the prevention and treatment of drug-resistant bacteria infections are urgently needed.

In this context, the rapid development of nanomaterials as potential biosensors (Chen et al., 2017) and antibacterial agents (Wang Y. et al., 2020), has attracted enormous attention in recent years, owing to their unique physiochemical attributes. By appropriately integrating with the bacteria-specific recognition elements, numerous nanomaterials especially metals (e.g., Au and Ag) and metal oxides/sulfides (*e.g.*, Fe_3_O_4_ and MoS_2_) with diversified morphologies, size, composition, structure, and surface charge, have been extensively utilized for bacterial detection based on the colorimetric/fluorescent/electrochemical responses (Gupta et al., 2021; Yang et al., 2021; Somvanshi et al., 2022). The diagnostic assays demonstrated appealing merits of simplicity, rapidness, and accuracy for early detection of pathogenic bacteria. For another, these biomaterials showed intrinsic or light-mediated bactericidal properties (e.g., photothermal and photodynamic activities) (Li et al., 2020; Huo et al., 2021; Fu et al., 2022), thereby offering opportunities to develop promising antibiotic-free antibacterial modalities. These photo-responsive therapeutic modalities involving photothermal/photodynamic therapies (PTT/PDT), by generating high temperatures from the light-active nanomaterials (e.g., Au and Ag nanoparticles) with photothermal conversion activity (Goncalves et al., 2020; Anuthum et al., 2022), or producing toxic reactive oxygen species (ROS) by irradiation (Qi et al., 2019; Zhu et al., 2022), are of special interest in bacterial killing, owing to their clean, non-invasive, and remotely controllable nature. However, despite their good prospect, mono-modal diagnosis or therapy often affords limited efficiency; meanwhile, the materials with only single component are often less functional and difficult to achieve both detection and killing of bacteria, which may restrict their potential for both laboratory research and biomedical industry.

To construct metal-based hybrid nanostructures (e.g., alloy, core-shell and supported heterostructures) is one of effective strategies for enhancing their functionalities (Park et al., 2016; Liu et al., 2021; Xie et al., 2022). The hybridization of multiplex components, via the various wet-chemical synthetic routes, could integrate their inherent functions so as to meet the practical requirements of diagnosis and therapy. A good example is the Au-Ag alloy nanoplates obtained through Galvanic exchange and Kirkendall growth (Qian et al., 2016), which displayed excellent SERS response for sensitive detection of the disease-related peptide (*β*-amyloid), as well as tunable NIR photothermal effect. In another interesting study (Zhu et al., 2020), by the *in situ* growth of Prussian blue (PB) on the Au nanoparticles (NPs) in liquid phase, the as-obtained Au@PB core-shell NPs have simultaneously shown good SERS response, enhanced magnetic resonance imaging (MRI) capacity, and photothermal/photodynamic activities, thereby allowing the SERS/MRI-guided tumor diagnosis and phototherapy. Notably, SERS could provide a simple, rapid yet sensitive testing technique based on molecular vibrations, which could amplify the normal Raman signal by tens of thousands of times through enhanced substrates, greatly raising the amplitude of the spectra (Lee et al., 2019). Meanwhile, due to intrinsic Raman response of PB in the biologically silent region (Yin et al., 2017; Li et al., 2019), it could avoid the overlap of characteristic peaks of common label molecules (e.g., 4-ATP and R6G) with the fingerprint region of the organism, largely reducing the interference in the spectroscopic analysis. The PB NPs could also be utilized as a type of catalytically active nanomaterials mimicking enzymes (namely, nanozyme), which have found broad applications in biological detection based on the colorimetric reaction by the peroxidase-mimic catalysis (Farka et al., 2018; Wang Z. et al., 2020), with many fascinating advantages (e.g., cost-effectiveness, easy preparation, high environmental stability, and flexible surface modifications). However, despite promise of metallic heterostructures in biomedical areas, they are less explored for integrative bacterial detection and treatment. To rationally design and controllably construct the desired nanostructures with extended versatilities, that organically combine accurate detection and efficient therapy, with a comprehensive understanding of synergistic mechanism, remains a challenge.

On the other hand, considering ever-increasing requirements of practical applications, the exploration of novel metallic nanostructures with enhanced functionalities has attracted increasing interest in biomedical areas recently (Guselnikova et al., 2022). The gold nanobipyramids, as a type of emerging and versatile plasmonic NPs, have shown outstanding SERS response due to their sharp tips which remarkably enhance the local electric field (Chow et al., 2019), thus providing a superior substrate to construct advanced SERS biosensors. They were also utilized as efficient photothermal materials due to their strong localized surface plasmon resonance (LSPR) absorption in the NIR region (Lemcoff et al., 2023), as well as potential support matrices to construct metallic heterostructures, for example, the Au@Ag core-shell nanorods by *in situ* overgrowth of the Ag atoms (Zhu et al., 2016). Meanwhile, Ag nanomaterials exhibited intrinsic antibacterial activities by releasing Ag^+^ ions to deactivate bacterial substances, which had a broad-spectrum action (Zhou et al., 2022). Therefore, by properly integrating with Ag and PB, the functionalities of the gold nanobipyramids could be expected to be extended, from SERS/nanozyme activities for bacterial detection, to the combined PTT/PDT effects and Ag^+^ ions release for sterilization.

**SCHEME 1:**
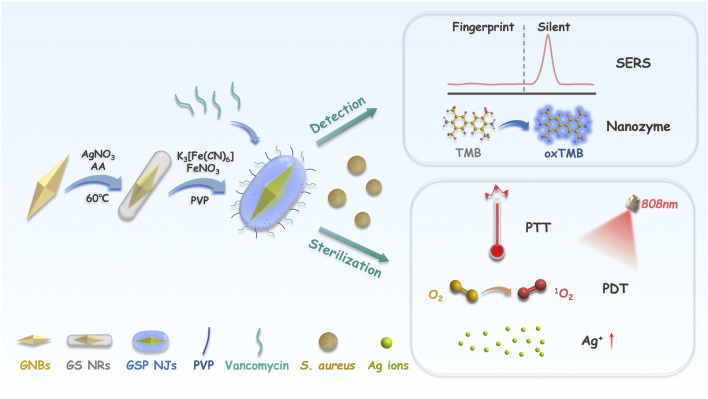
Illustration of the construction of the GSP NJs and applications. The GSP NJs were synthesized *via* template-etching, and the functionalization of vancomycin endowed them with targeting to the *S. aureus*. For bacterial detection, the response of SERS and the absorbance of *ox*TMB depended on the concentration of the captured bacteria. For bacterial elimination, the synergistic effect of heat, singlet oxygen and Ag ions produced by the NJs under irradiation caused bacterial death.

Herein, we report a novel strategy for dual bacterial detection and sterilization, by constructing the core-shell nanostructures composing of gold-silver-PB nanojujubes (GSP NJs) *via* a template method (Scheme 1). To present the synthesis, the gold nanobipyramids were used as a template for the growth of Ag atoms to form the core-shell Au@Ag nanorods, then the outer Ag atoms were reacted with K_3_ [Fe(CN)_6_] and FeNO_3_ to yield PB, in the presence of polyvinyl pyrrolidone (PVP) as a stabilizer. The as-formed GSP NJs showed controllable morphology, uniform size, and fine dispersibility. The functionalization of vancomycin on GSP NJs (named GSPv NJs) further endowed the material with good targeting to the model bacteria *S. aureus*. Interestingly, the Ag layer not only acted as a sacrificed template for the formation of outer PB, but served as the source of Ag^+^ ions, which were accommodated in the PB layer. The as-obtained GSP NJs showed strong bio-silent SERS signal and high peroxidase-like activity, thereby making them useful for flexible and accurate dual-mode bacterial detection. Meanwhile, they exhibited excellent PTT and PDT activities, as well as enhanced Ag^+^ ions release by near-infrared (NIR) light irradiation for bacterial killing. The work could provide new insights into the design of versatile metallic heterostructures for bacterial detection and sterilization.

## 2 Results and discussion

### 2.1 Synthesis and characterization

**FIGURE 1:**
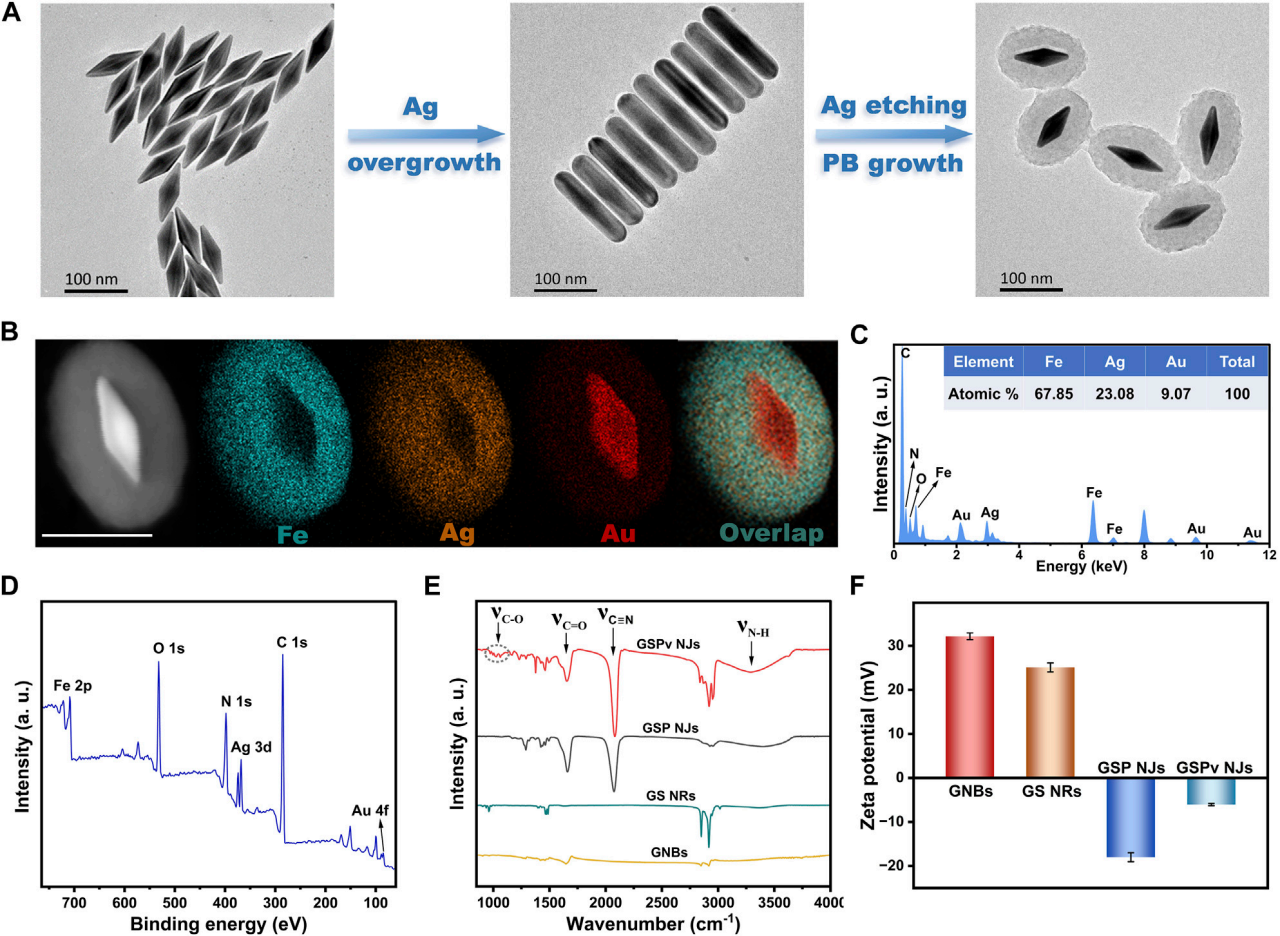
Synthetic illustration and characterization of the GSP NJs. **(A)** Illustration of the synthesis of the GSP NJs captured by TEM. **(B)** HAADF-STEM image and elemental maps of the GSP NJs. Scale bar: 100 nm. **(C)** EDS spectrum and **(D)** XPS survey spectrum of the GSP NJs. Comparison of **(E)** IR spectra and **(F)** Zeta potential of the GSP NJs with other materials.

The GSP NJs were synthesized by a two-step template etching, which underwent successive morphological evolutions: from initial gold nanobipyramids (GNBs), to gold-silver nanorods (GS NRs), and to final gold-silver-PB nanojujubes (Figure 1A). First, the GNBs were synthesized through a seed growth method (see details in Experimental section). By varying the feeding of the seeds, a series of the GNBs with different aspect ratios (ARs) were obtained (Supplementary Figures S1A-D). They had a characteristic LSPR absorption peak in the NIR region, which was red-shifted as the AR increased (Supplementary Figure S1E). Here, the GNBs with an AR of ∼3.5 (Supplementary Figure S1B) was chosen as the cores to construct the GSP NJs, whose wavelength of LSPR (802 nm) was close to that of the commonly used PTT/PDT laser (808 nm) and typical Raman excitation light (785 nm). Then, the Ag atoms were grown on the surface of the GNBs, through the reduction of AgNO_3_ by ascorbic acid (AA). The continuous growth of Ag atoms afforded the Au@Ag nanorods, evidenced by transmission electron microscopy (TEM, Supplementary Figure S2). The typical GS NRs with an AR of ∼3.8 were selected as a sacrificial template for further synthesis. Subsequently, by using K_3_ [Fe(CN)_6_] and FeNO_3_ as the etchants, the as-formed Ag shell layers were etched away (Supplementary Figure S3), accompanying the generation of PB based on the following equation: 3Ag + 3K_3_Fe(CN)_6_ + 4Fe(NO_3_)_3_ → Fe_4_ [Fe(CN)_6_]_3_ + 3AgNO_3_ + 9KNO_3_ (Shen et al., 2014).

The core-shell nanostructures of the as-obtained GSP NJs were proved by high-angle annular dark-field scanning transmission electron microscopy (HAADF-STEM). From one representative GSP NJ with a shell thickness of 35 nm, Figure 1B showed its STEM image and corresponding elemental maps, in which the Ag elements remained after etching, with even distribution in the PB shell layer. A Fe/Ag/Au atomic ratio of 67.85 : 23.08: 9.07 was further obtained by energy dispersive X-ray spectrometry (EDS; Figure 1C). Since no obvious larger-sized particles were observed in the high-resolution TEM (HRTEM) images (Supplementary Figure S4), the Ag species could be existed in the form of ultra-small clusters and ions. The composition of the metals was again attested by X-ray photoelectron spectroscopy (XPS, Figure 1D). By the high-resolution peak-fitting, the binding energy (BE) peaks of Au 4f (85.4 and 89.0 eV) were attributed to the metallic Au (Supplementary Figure S5A) (Hang et al., 2019), while two pairs of peaks in the Ag 3d spectrum (Supplementary Figure S5B) suggested the co-existence of Ag^0^ (368.3 and 374.3 eV) and Ag^+^ (367.8 and 373.9 eV) (Shang et al., 2010), in line with the finding by HRTEM. Besides, a mixed Fe^2+^ (708.6 and 721.7 eV) and Fe^3+^ (710.6 and 723.8 eV) species were also observed in Fe 2p spectrum (Supplementary Figure S5C) (Wang et al., 2022).

To enhance dispersibility of the NJs, PVP was introduced in their synthesis, considering its good hydrophilicity and binding affinity to PB (Hu et al., 2012). Further functionalization by vancomycin could provide specificity towards *S. aureus*. From Figure 1E, the PVP coating was verified by absorption at 1665 cm^-1^ (C=O stretching) in the IR spectrum, while the new bands at 3100–3500 cm^-1^ (N-H stretching) and 960–1130 cm^-1^ (C-O stretching) indicated the existence of vancomycin. Besides, a strong absorption at 2080 cm^-1^ (C≡N stretching) for the cyanide proved the formation of PB, where the assistance of PVP at a moderate concentration (5 mg/mL) allowed a well-defined shell layer in the products with best monodispersibility (Supplementary Figure S6). The functionalized species were also confirmed by comparing the surface charge before and after modification (Figure 1F), in which PVP gave an obvious decrease in Zeta potential of the NRs, while vancomycin led to a slight increase in Zeta potential of the NJs. Collectively, the GSP NJs were successfully constructed and functionalized.

**FIGURE 2:**
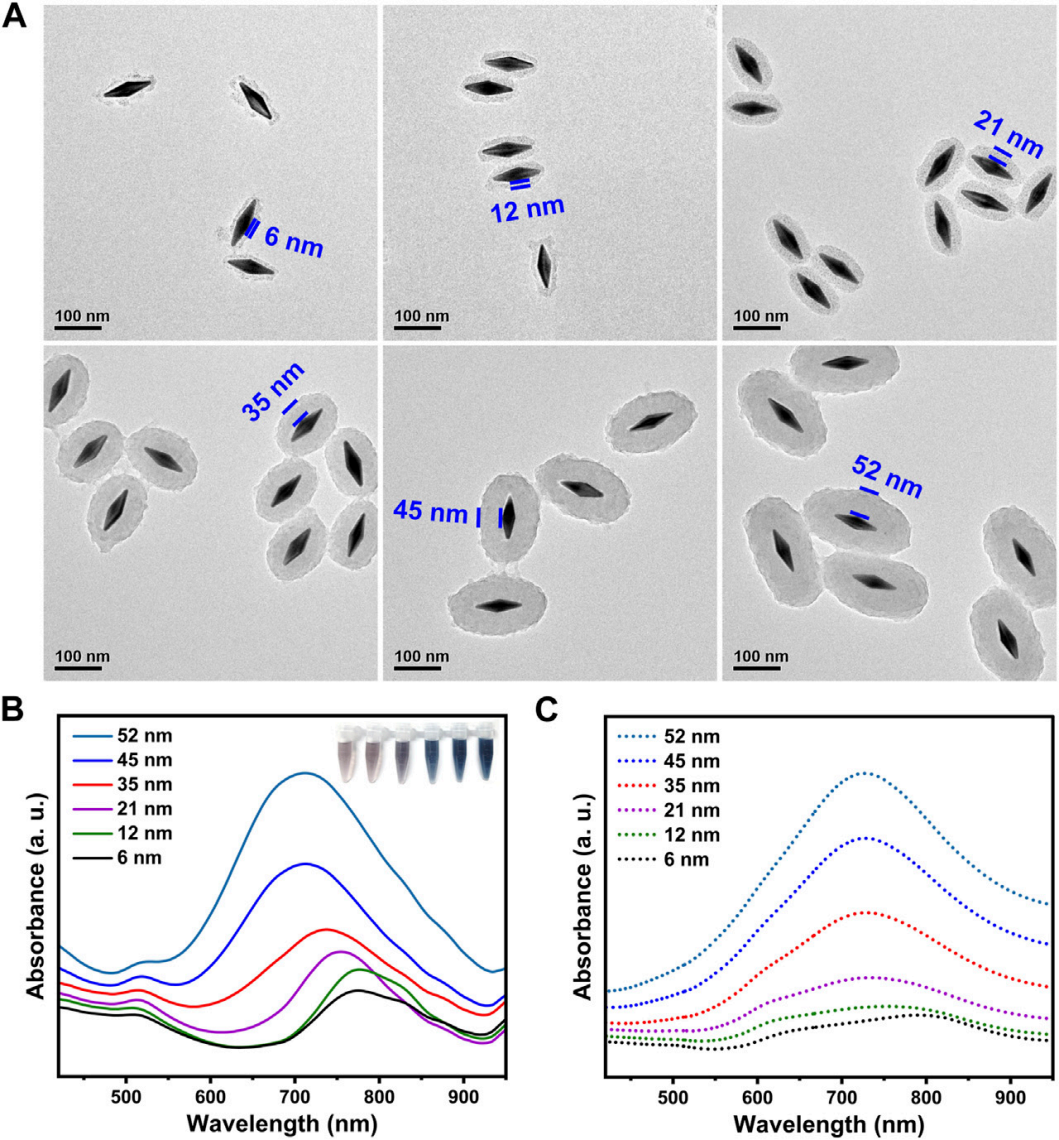
Characterization of the GSP NJs with different shell thickness. **(A)** TEM images. **(B)** Ultraviolet-Visble-NIR (UV-VIS-NIR) absorption spectra and corresponding photographs. **(C)** The calculated absorption spectra.

One of attractive advantages of present synthesis lies in the controllable shell thickness. By simply adjusting feeding of AgNO_3_ and the etchants, the shell thickness could be finely adjusted, thus providing an ideal core-shell model of ternary nanojujubes for probing the structure-property relationship. Figure 2A showed TEM images of a series of the GSP NJs with different shell thickness, from 6 nm to 52 nm. They had a strong absorption peak in the NIR region, which was gradually blue-shifted to the characteristic peak of PB (around 720 nm) as the shell got thicker (Figure 2B); correspondingly, the peak intensity increased and the colloidal color was changed from brown to blue. By changing the geometry of the NJs, one can modulate their absorption. The simulation shown in Figure 2C was well matched with the above experiment results. Overall, the synthesis showed merits of easily available reagents, mild reaction conditions, convenient operations, as well as controllable morphology and size of the materials. Here, the GSP NJs with a moderate thickness of shell (35 nm) were selected as a model for further studies. As can be seen from the Supplementary Figures S7A-B, the GSP NJs retained good morphology and dispersibility after 1 month of storage compared to the freshly prepared NJs. Moreover, there was no significant change in the absorbance at 720 nm (Supplementary Figure S7C). Such stabilities of both morphology and photo-responsive properties of the NJs could offer possibilities for long-term studies on bacterial detection and elimination.

### 2.2 SERS and enzyme-like properties

**FIGURE 3:**
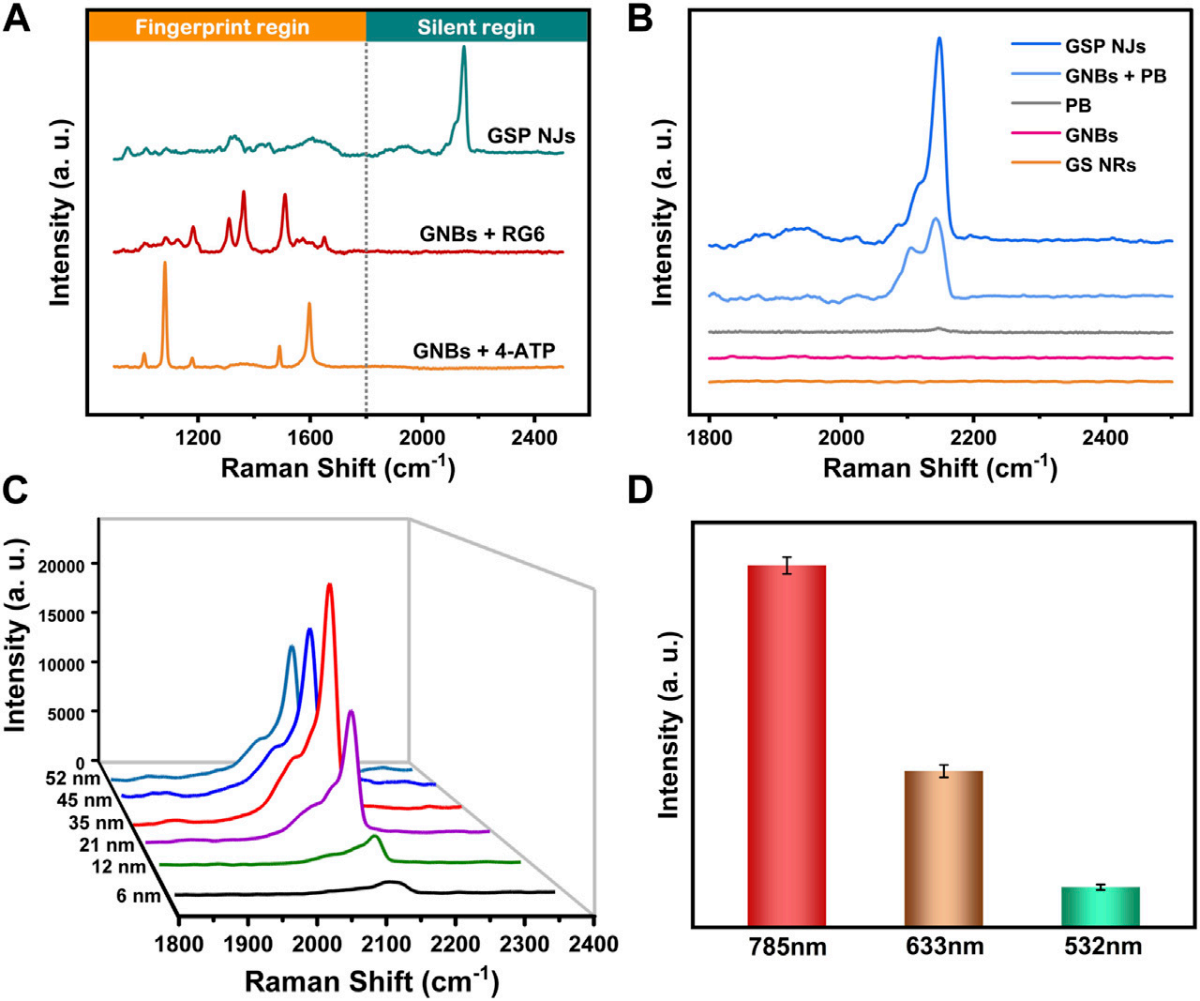
SERS spectra of the GSP NJs. **(A)** The signal responses of the GSP NJs, RG6 and 4-ATP enhanced by the GNBs in silent and bio-fingerprint region. **(B)** Analysis of enhancing ability of GSP NJs through the comparison between PB, GNB, mixture of GNBs and PB, GSP NJs and GS NRs. **(C)** Variation of signal strength with the GSP NJs shell thickening. **(D)** The effect of the wavelength of Raman excitation laser on the intensity of the peak (2150 cm^-1^) of the GSP NJs.

Early detection of pathogenic bacteria outbreaks is one key step to prevent massive infections. Compared with conventional diagnostic methods based on plate counting (Kanagawa et al., 2018) or polymerase chain reaction (Wang et al., 2016), SERS could provide a rapid and sensitive spectroscopy technique for bacterial detection, but the direct output of the signal is often complex and weak, which generally requires modification of an appropriate label molecule on the substrate. Unfortunately, the characteristic peaks of commonly used label molecules usually overlap with the biological fingerprint region between 800–1800 cm^-1^ (Zong et al., 2018; Zhou et al., 2020). In our study, since PB had a characteristic peak (2150 cm^-1^) in the bio-silent region (Figure 3A), the signal interference from organisms was avoided. Moreover, the signal intensity depended on the amplitude of the substrate enhancement; without GNBs, the peak of PB itself was faint (Figure 3B). The signal intensity was also affected by the affinity between PB and GNBs. Since PB could be stably fixed on the GNBs by *in situ* growth, the GSP NJs gave a stronger and sharper peak in the SERS spectrum relative to the mixture of GNBs and PB.

The effects of two parameters (PB shell thickness and excitation light wavelength) on SERS response were further investigated. From Figure 3C, as the shell thickness increased from 6 nm to 35 nm, the SERS signal gradually became stronger because of more label molecules; however, when the shell thickness was larger than 35 nm, the signal became weaker, since the LSPR absorption from the GNBs might be hindered. Meanwhile, by comparing various excitation light wavelengths (532, 633 and 785 nm), the largest wavelength selected (785 nm) displayed the highest enhancement (Figure 3D), closest to the LSPR absorption peak of the GNBs. The signal reproducibility of the GSP NJs was further evaluated. After testing at 12 randomly selected positions of the liquid sample, the relative standard deviation (RSD) was measured to be 2.78% (Supplementary Figure S8), demonstrating good reproducibility.

**FIGURE 4:**
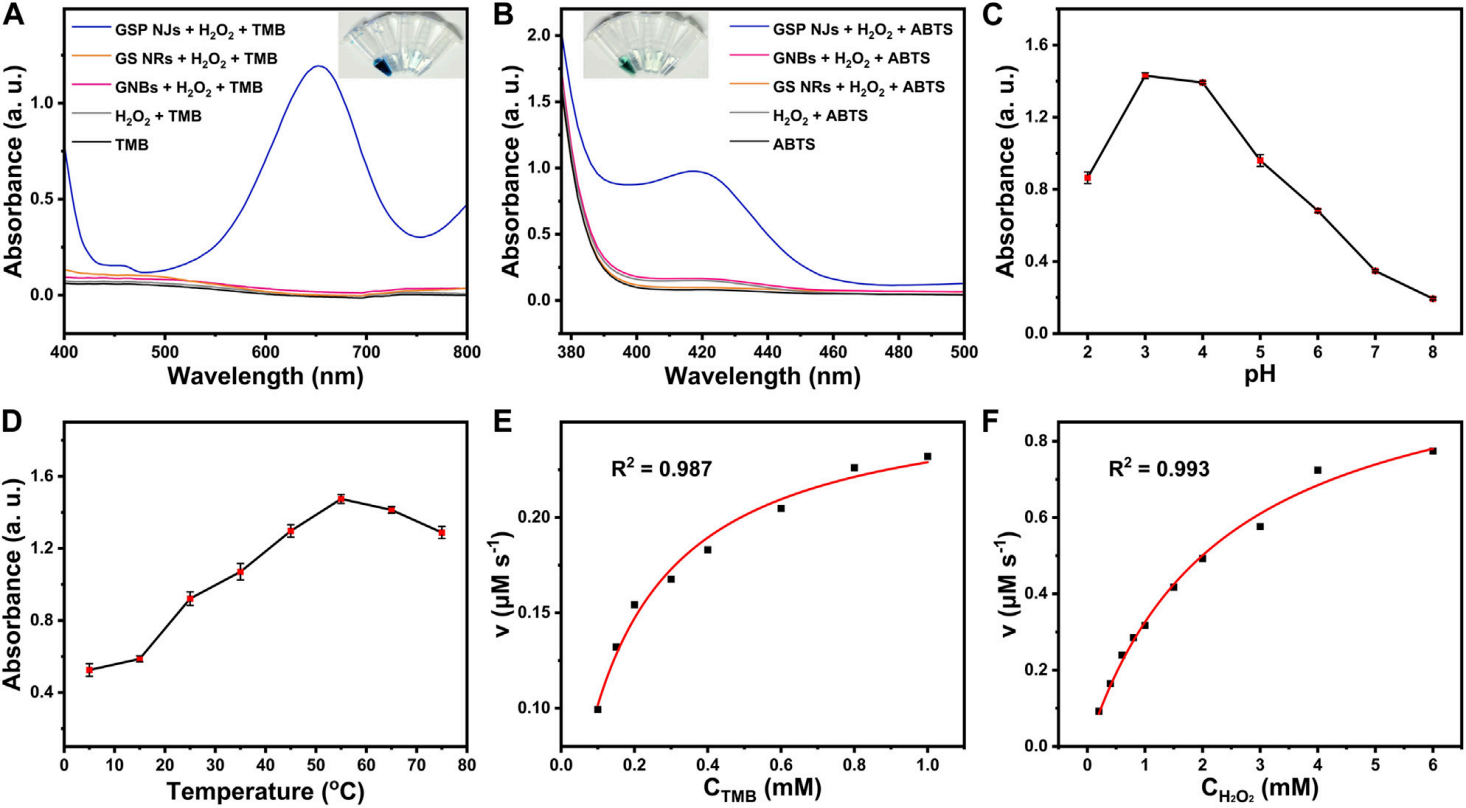
Peroxidase-like activities of the GSP NJs. UV-VIS spectra and the corresponding color changes of the solutions for the catalytic oxidations of **(A)** TMB and **(B)** ABTS by the GNBs, GS NRs and GSP NJs, respectively. The effect of **(C)** pH and **(D)** temperature on the absorbance of oxidized TMB at 652 nm by the GSP NJs. Catalytic kinetics of the GSP NJs analyzed by Michaelis-Menten model toward substrate of **(E)** TMB and **(F)** H_2_O_2_.

Besides, the peroxidase-like activity of the GSP NJs was also tested. As shown in Figures 4A,B, in the presence of H_2_O_2_, the NJs catalyzed oxidations of colorless 3,3′,5,5′-tetramethylbenzidine (TMB) and 2,2′-azino-di-(3-ethylbenziazobine sulfonate-6) (ABTS) to produce the blue and green products, with maximum absorption at 652 nm and 420 nm, respectively, whereas the solutions containing GS NRs and GNBs did not show much changes in the color of the reaction solutions. Moreover, the NJs showed good catalytic activity in a wide range of pH 2-8 (Figure 4C) and temperature of 5–75°C (Figure 4D). Under the optimal condition (pH 3 and 55°C), the kinetic experiments were further conducted. The collected kinetic data, by varying the concentration of the substrates, were well fitted by the Michaelis-Menten model (Figures 4E,F) and Lineweaver-Burk model (Supplementary Figure S9), which gave two important kinetic parameters, *i.e.*, the maximum reaction rate (*v*_max_) and affinity constant (*k*_m_). In principle, the former characterizes the catalytic capacity of an enzyme, the larger the value the more active the enzyme, while the latter is the concentration of the substrate at 1/2 *v*_max_ and reflects the affinity of an enzyme towards the substrate, the smaller the value the higher the affinity. As listed Supplementary Table S1, compared to horseradish peroxidase (HRP) (Gao et al., 2007), the GSP NJs gave a larger *v*_max_ and a lower *k*_m_, indicating higher activity and better affinity towards TMB or H_2_O_2_.

Previous studies have revealed two types of reaction pathways in peroxidase-mimic catalysis: 1) Fenton-like way that generates hydroxyl radicals (•OH) (Tao et al., 2015; Fang et al., 2018), and 2) electron transfer between the enzyme and the substrate (Wu et al., 2018; Cai et al., 2020). However, by electron spin resonance (ESR) spectroscopy using 5,5-dimethyl-1-pyrroline *N*-oxide (DMPO) as a trap, no •OH radicals were detectable during the catalytic process (Supplementary Figure S10A). To verify this finding, the oxidation of terephthalic acid (TA) was conducted, as it can be oxidized by •OH radicals to yield fluorescent product, with a typical peak at about 430 nm (Hassanzadeh et al., 2018). However, after addition of the GSP NJs and H_2_O_2_, the fluorescence intensity at this peak decreased compared with the control experiments (Supplementary Figure S10B), thereby ruling out the •OH generation. Then, the electron transfer process was further proved by the oxidation of reduced cytochrome C (RCC), since RCC is an active electron donor (Fu et al., 2021). From Supplementary Figure S11, when the GSP NJs were present, the typical absorption peaks of RCC at 519 and 549 nm disappeared while a new peak appeared at 530 nm, corresponding to the oxidized cytochrome C. The result indicated the material accepted electrons from the substrate.

On the other hand, the electronic structure of the GSP NJs was analyzed by XPS. Compared with the Au 4f_7/2_ core level BE (84.1 eV) in the GNBs (Supplementary Figure S12), the NJs showed a positive BE shift (1.3 eV), indicating that the electronic structure of the Au atoms was regulated, in which electrons transferred from the Au atoms to outer shells. Such an electronic migration induced the formation of electron-rich shells, beneficial to the oxidation on the surface of the GSP NJs. Therefore, with modification of electronic structure of the Au cores, the nature of peroxidase-like catalysis of the GSP NJs was attributed to the efficient electron transfer capacity.

### 2.3 Bacterial detection

**FIGURE 5:**
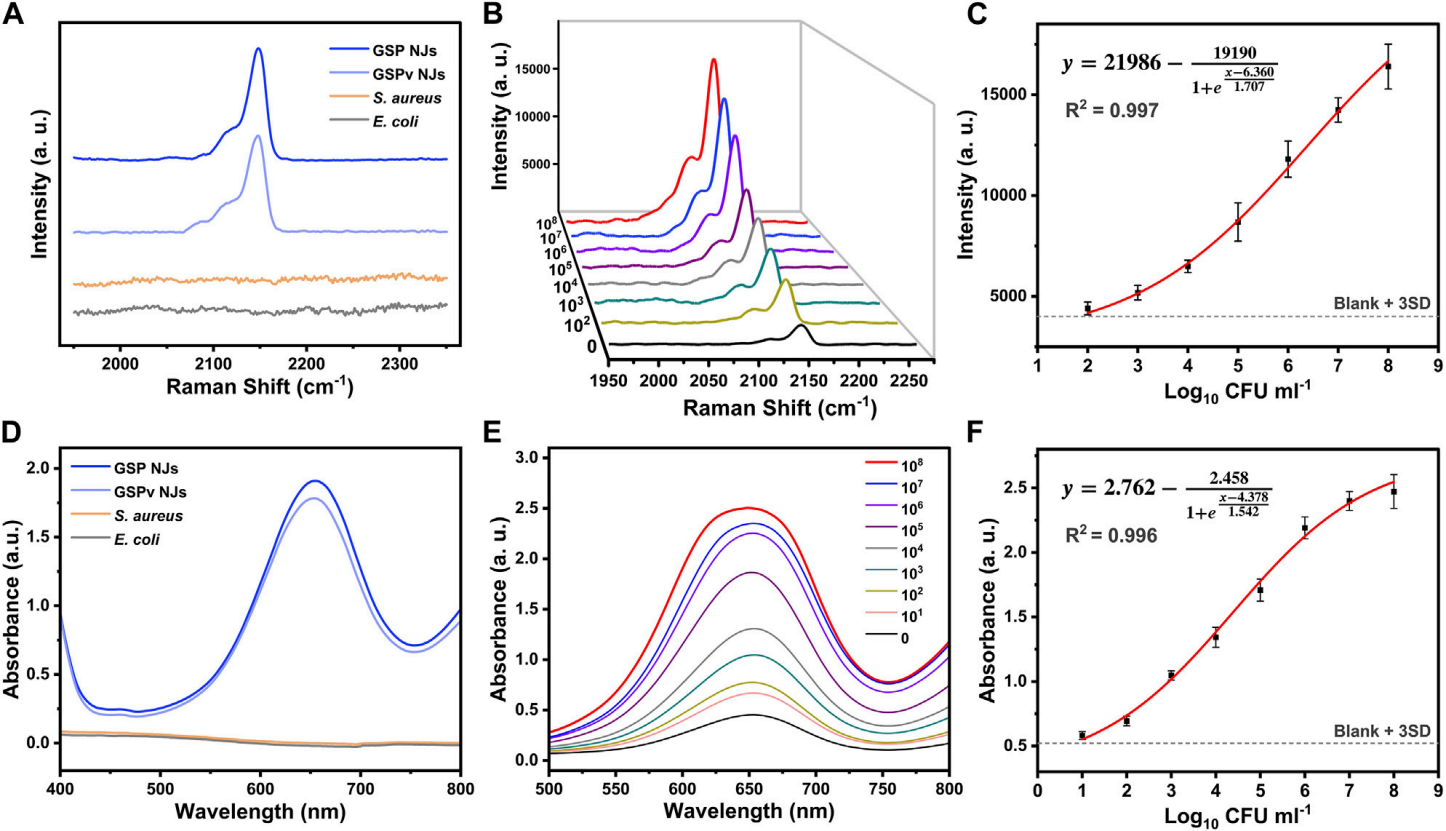
Bacterial detection from the **(A–C)** SERS and **(D–F)** nanozyme modes. **(A, D)** The effect of bacteria and vancomycin on the signal. **(B, E)** Detection of *S. aureus* at different concentrations using different methods. **(C, F)** The relationship between signal intensity and logarithmic concentration of *S. aureus* fitted by the Sigmoidal model.

Considering Raman response and peroxidase-like activity, the GSP NJs could provide two modes of detections of SERS and nanozyme. For the former, possible influences of vancomycin and bacteria on the SERS signal were first investigated. From Figure 5A, the characteristic peak of PB (2150 cm^-1^) remained almost unchanged after modification of vancomycin, while the bacteria did not show a visible peak in this region, thereby excluding their interference with SERS signal. Then, SERS detection was performed for bacteria in the concentration range of 0–10^8^ CFU/mL. From Figure 5B, as the concentration of bacteria increased, the peak intensity increased. The relationship between peak intensity and logarithmic concentration could be fitted well with the Sigmoidal model (Figure 5C), which afforded a high coefficient of correlation (R^2^ = 0.997). Meanwhile, for nanozyme mode, potential interference of vancomycin and bacteria with peroxidase catalysis was studied. From Figure 5D, the vancomycin coverage only caused a slight decrease in absorbance of oxidized TMB (*ox*TMB) at 652 nm, and bacteria themselves did not show any catalytic activity. As the concentration of bacteria gradually increased, absorbance of *ox*TMB steadily increased (Figure 5E), and the relationship between logarithmic concentration and absorbance was also well in accordance with the Sigmoidal function, giving a large value of R^2^ of 0.996 (Figure 5F).

The detection of limit (LOD) was further determined by the IUPAC standard method, which is the concentration corresponding to the blank signal intensity plus three times the standard deviation. According to the fitting equation, the LOD values for the SERS and nanozyme modes were 56 and 6 CFU/mL respectively. Compared with other assays reported, these modes showed a shorter detection time and a lower LOD (Supplementary Table S2). Meanwhile, compared with SERS mode, nanozyme mode required addition of substrates for the catalytic reaction, but afforded an active response and a lower LOD. Therefore, the dual activities of the GSP NJs could offer opportunities to flexibly select an appropriate detection mode for the practical requirements, and the combination of these modes could bring more accurate results. Besides, the assays also demonstrated high specificity. Compared with Gram-negative bacteria like *E. coli*, *S. aureus* showed higher responses for both SERS and nanozyme modes (Supplementary Figure S13).

### 2.4 Photo-responsive antibacterial activities

**FIGURE 6:**
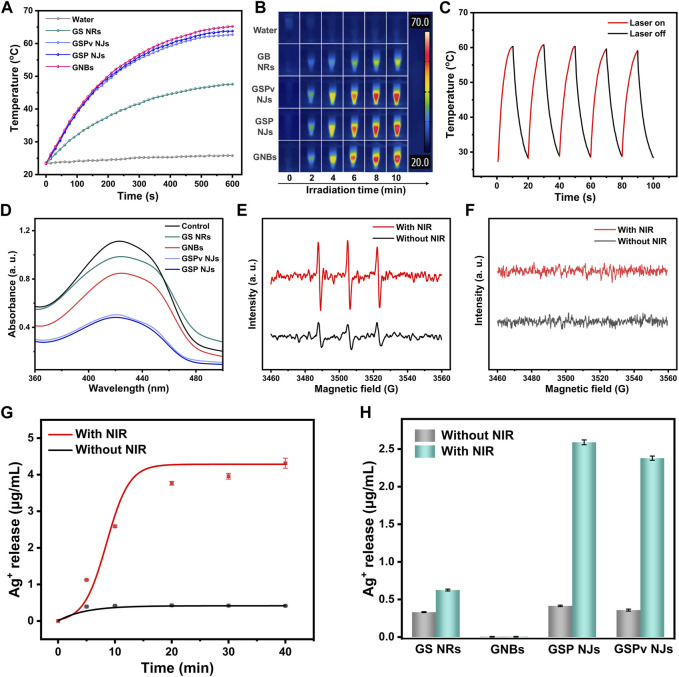
Photo-responsive antibacterial activities of the as-prepared materials. **(A)** Photothermal heating curve under NIR irradiation for 10 min. **(B)** The corresponding real-time infrared thermal image. **(C)** Photothermal stability of the GSP NJs for 5 cycles of heating and cooling. **(D)** PDT induced ROS measured by the UV-VIS spectra of DPBF. **(E)** ESR spectra of ^1^O_2_ radicals generated by the GSP NJs using TEMP as a trap. **(F)** ESR spectra of possible O_2_^•−^ or •OH radicals generated by the GSP NJs using DMPO as a trap. **(G)** Time-dependent Ag ions release curve of the GSP NJs treated with or without NIR irradiation. **(H)** Statistics of Ag ions release from different materials with or without NIR irradiation for 10 min.

Since both the GNBs and PB had NIR photothermal activities, the GSP NJs could inherit their natures. An 808 nm laser was then chosen as the irradiator, which has benefits of deep tissue penetration, less side-effect and high absorption coefficient (Jiang et al., 2022). From Figures 6A, B, under irradiation (0.8 W/cm^2^), the GSP NJs (80 μg/mL) gave an obvious increase in the temperature from 23°C to 64°C in 10 min, comparable to the GNBs (65°C) and much higher than that of GS NRs (47°C). Moreover, there was no obvious decrease in the photothermal effect after vancomycin modification. Such photothermal property was closely relied on the concentration of the NJs and power density of irradiation, in which a higher concentration or power density led to a higher temperature (Supplementary Figure S14). Besides, the photothermal stability of the GSP NJs was investigated. After 5 cycles of heating and cooling, the highest temperature dropped by only 1.5°C (Figure 6C), indicating that they had a good temperature tolerance.

We also studied whether the GSP NJs could display photodynamic activity, inducing the generation of singlet oxygen (^1^O_2_) in air under NIR irradiation. The possible ^1^O_2_ was probed using 1,3-diphenylisobenzofuran (DPBF) as a trapping agent, as it can be oxidized by ^1^O_2_, resulting in a decline of absorbance at about 425 nm (Zhu et al., 2021). From Figure 6D, with GSP NJs, the absorbance of DPBF was reduced significantly under irradiation, indicating that they had photodynamic activity. Compared with the GS NRs and GNBs, the GSP NJs exhibited better activity, while modification of vancomycin did not cause an obvious decrease in the absorbance. The generation of ^1^O_2_ radicals by the GSP NJs was also verified by ESR with 2,2,6,6-tetramethylpiperidinyl-*N*-oxide (TEMP) as a trap (Figure 6E). Meanwhile, other possible ROS (*e.g.*, •OH and O_2_^•−^) were investigated by ESR using DMPO as a probe. However, these radicals were not detectable (Figure 6F). Thus, the GSP NJs induced O_2_ to ^1^O_2_ by the PB shells under irradiation. This photodynamic response, together with photothermal activity, potentially allowed the NJs to achieve combined PTT-PDT therapies. Notably, such integrated photo-responsive modalities, involving *in situ* growth of PB as an intrinsically light-active substance, was different from common PDT or PTT-PDT therapies, which generally involve loading of photosensitizers on the materials with complicated modification (Zhou et al., 2018).

Besides, the Ag-based antibacterial agents often require a construction of complex porous structures and suffer from low Ag^+^ ions release (Cao et al., 2020; Xu et al., 2021). Interestingly, as a typical metal-organic framework (MOF) with porous structure (Kong et al., 2015), PB could act as an efficient reservoir of Ag ions or clusters, which were further released into the aqueous phase. From Figure 6G, without irradiation, the GSP NJs only released 0.41 μg/mL of Ag^+^ in 20 min; however, when irradiation was introduced, the value was increased to 3.76 μg/mL. This photo-promoted ion release may be due to an increase in temperature by the PTT activity, which not only promoted oxidation of the highly reactive Ag clusters to ions (4Ag + O_2_ → 2Ag_2_O, Ag_2_O+ 2H^+^→ 2Ag^+^ + H_2_O) (Guo et al., 2022), but accelerated the release of Ag^+^ ions inside (Mo et al., 2015). For comparison, the GS NRs showed a lower release of Ag^+^ under irradiation, owing to their compact structure and poorer PTT activity (Figure 6H). Notably, the Ag^+^ release was not affected by the modification of vancomycin significantly. These results suggested the unique nanostructure of the GSP NJs endowed them with efficient PTT/PDT activities and photo-promoted Ag^+^ release ability.

### 2.5 Antibacterial studies

**FIGURE 7:**
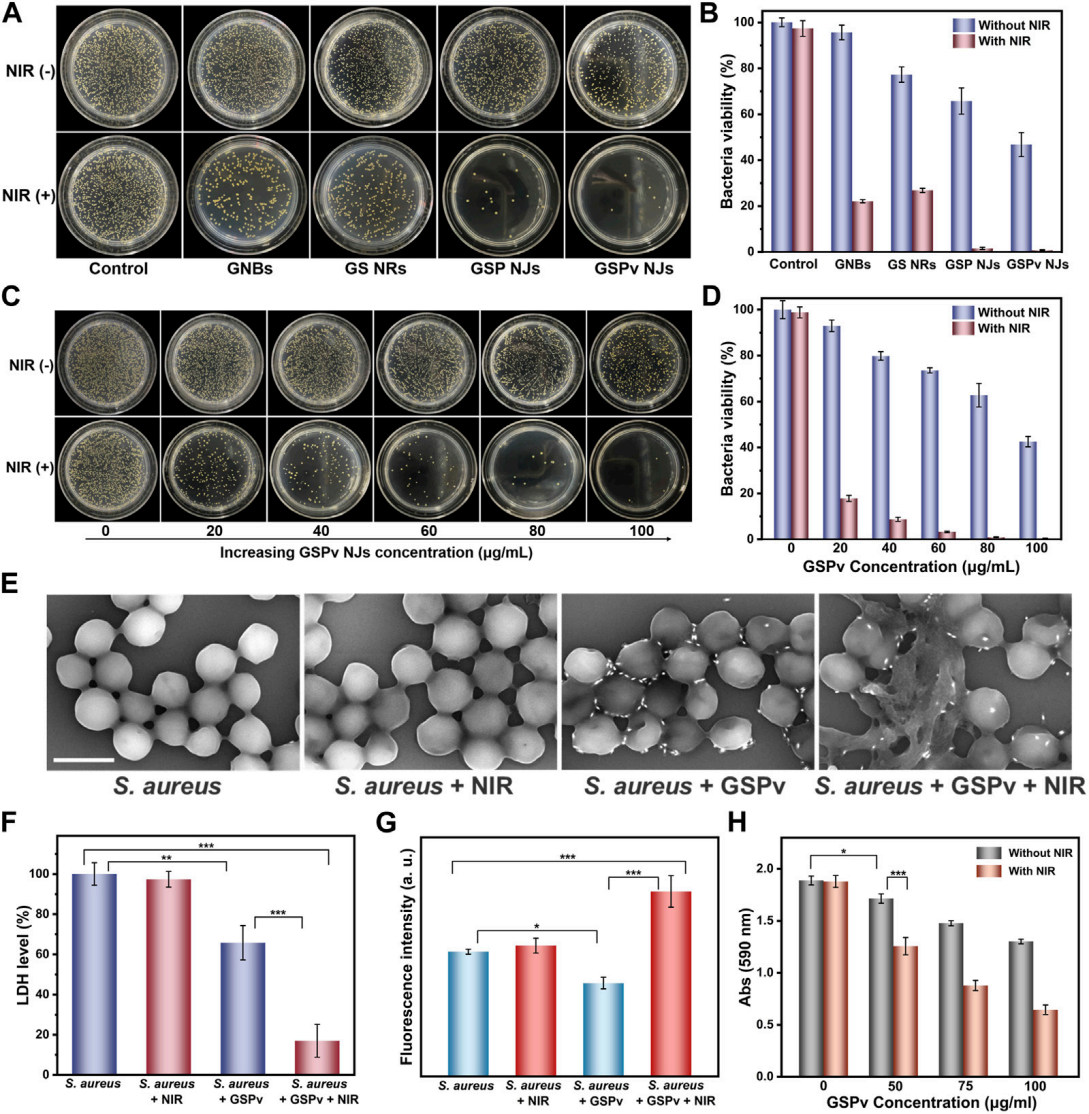
Bacterial killing by the NJs. **(A)** Photographs of *S. aureus* colonies incubated on agar plates treated by the materials (80 μg/mL) in the presence or absence of NIR irradiation (0.8 W/cm^2^) and **(B)** the corresponding statistics of colonies on the plates. **(C)** Photographs of bacteria treated by different concentrations of the GSPv NJs in the presence or absence of NIR irradiation (0.8 W/cm^2^) and **(D)** the corresponding statistics of colonies on the plates. Damage of *S. aureus* structure under different treatments of GSPv NJs and NIR irradiation, characterized by **(E)** SEM (1 μm of the scale bar) and **(F)** LDH leakage. **(G)** Oxidative damage of bacteria analyzed by DCFH-DA fluorescence spectrum. **(H)** Biofilms elimination capacity of the GSPv NJs analyzed by the 590 nm absorbance of crystal violet. Error bars indicate means ± standard deviations (*n* = 3 biologically independent samples). Statistical significance was analyzed by one-way ANOVA with Turkey test: *p < 0.05, **p < 0.01 and ***p < 0.001.

Encouraged by the above findings, the GSP NJs could be effective for sterilization. From Figures 7A, B, without NIR irradiation, the GSP NJs exhibited better bacterial elimination than the GNBs and GS NRs, which was ascribed to the more Ag^+^ release. By introducing NIR irradiation, the GSP NJs showed a 98.4% of sterilization efficiency, higher than that of the GNBs (77.9%) and GS NRs (73.1%). Moreover, the antibacterial ability of GSPv NJs was further enhanced because of the modification of vancomycin, ultimately leading to death of 99.2% of bacteria.

Numerous studies (Jia et al., 2017; Gwon et al., 2020) have shown that the antibacterial activities of nanomaterials were concentration-dependent. Similar observations were obtained by the GSPv NJs; the higher the concentration, the greater the activity (Figures 7C,D). It was found 100 μg/mL of the GSPv NJs were able to kill over 99.9% of bacteria with NIR irradiation for 5 min, demonstrating high sterilization efficiency. The GSPv NJs could also kill Gram-negative bacteria like *E. coli*. From the Supplementary Figure S15, when *E. coli* was incubated with the GSPv NJs, the bacterial growth was largely inhibited and almost all died after further NIR irradiation. Thus, the GSPv NJs exhibited their broad-spectrum antibacterial behavior. In addition, the normal cells maintained 83% of viability when incubated with the NJs with a high concentration of 100 μg/mL for 24 h (Supplementary Figure S16), indicating that the NJs had good biocompatibility.

The antibacterial mechanism of the GSPv NJs was further investigated. First, the morphologies of the bacteria were characterized by SEM. From Figure 7E, the normal *S. aureus* appeared mostly as a spherical shape and NIR irradiation did not change its shape. However, when incubated with the GSPv NJs, the surface of the bacteria became flattened and invaginate. By further introducing irradiation, the morphology of bacteria was dramatically destroyed, with a significant leakage of cytoplasm, implying that their membrane structures were damaged. The result was also corroborated by the leakage of LDH, a peripheral membrane protein located on the cytoplasmic side (El-Kaliuoby et al., 2021). As shown in Figure 7F, after incubation with the GSPv NJs, about 34.2% of the bacterial LDH was leaked, which rose to 83.1% when irradiated. Thus, the GSPv NJs led to serious damage to bacterial membrane and greatly accelerated the death of bacteria.

Besides, the possible oxidative damage to the bacteria was also studied by quantifying the intracellular ROS level. In the assay, the internal oxidation of *S. aureus* was measured by using 2,7-dichlorodihydrofluorescein diacetate (DCFH-DA) a probe, as it can be oxidized by ROS to produce fluorescent DCF (Chen et al., 2019). As shown in Figure 7G, without NIR irradiation, the fluorescence intensity slightly decreased when the bacteria were incubated with GSPv NJs, probably due to ROS scavenging by PB (Zhang et al., 2016). On the contrary, after irradiation, the ROS levels increased significantly, above normal cellular levels. The rise of ROS came from two pathways (Roy et al., 2019): one being the cellular oxidative stress of the bacteria due to stimuli of high temperature and massive Ag^+^ ions, and the other acellular ROS being the ^1^O_2_ produced by the GSPv NJs. Therefore, both cellular and acellular ROS induced by the GSPv NJs under NIR irradiation caused oxidative damage to bacteria and promoted bacterial death.

Biofilms have been recognized as a challenge in bacterial elimination (Lencova et al., 2021). A biofilm is a sticky film layer formed by a large number of bacteria and extracellular polymers adhering to the surface of a tissue or implant, posing a barrier to the traditional therapeutic approaches. Considering superior antibacterial activities of the GSPv NJs, we studied whether they could be further applied in biofilm removal. In the assay, the biofilm level was quantified by the absorption of crystalline violet at 590 nm, a commonly used staining method for biofilms (Liu et al., 2020). As shown in Figure 7H, the absorption gradually decreased as the concentration of the GSPv NJs increased; by further introducing the NIR irradiation, the absorption became lower. The result indicated that the GSPv NJs were effective in biofilm degradation.

## 3 Conclusion

In summary, the gold-silver-Prussian blue nanojujubes have been successfully synthesized in the presence of PVP as a stabilizer and further functionalized with vancomycin. The as-obtained GSP NJs showed uniform morphology, controllable size, and good colloidal dispersibility. Interestingly, due to the incorporation of multi-components in the heterostructures, the NJs simultaneously displayed good SERS property and peroxidase-like activity, thereby providing the dual modes for bacterial detection, with virtues of rapidness, high sensitivity, and flexibility. The NJs also exhibited strong photothermal and photodynamic activities as well as the photo-promoted Ag^+^ release capabilities, which achieved a high antibacterial efficiency over 99.9%. Moreover, the NJs were effective in degrading biofilms. Therefore, the NJs showed potential in the prevention and treatment of bacterial infections. On the basis of the versatilities of the NJs, we believe that they could be useful for more different biomedical applications.

## 4 Experimental section

### 4.1 Chemicals and reagents

Chloroauric acid trihydrate (HAuCl_4_·3H_2_O), silver nitrate (AgNO_3_), ascorbic acid (AA), 4-aminothiophenol (4-ATP) and terephthalic acid (TA) were provided by Alfa Aesar. Potassium ferricyanide (K_3_ [Fe(CN)_6_]), ferric nitrate nonahydrate (FeNO_3_·9H_2_O), sodium borohydride (NaBH_4_), 3,3′,5,5′-tetramethylbenzidine (TMB), dimethylsulfoxide (DMSO) and rhodamine 6G (R6G) were supplied by Acros. Hexadecyl trimethyl ammonium chloride (CTAC), vancomycin hydrochloride, Prussian blue, 5,5-dimethyl-1-pyrroline *N*-oxide (DMPO) and 2,2,6,6-tetramethylpiperidine (TEMP) were purchased from Innochem Co., Ltd. (Beijing, China). Hexadecyl trimethyl ammonium bromide (CTAB), polyvinyl pyrrolidone (PVP, Mw = 10,000) and 2′-azinobis-(3-ethylbenzthiazoline-6-sulphonate) (ABTS) were bought from Sigma-Aldrich. Hydrogen peroxide (H_2_O_2_) was supplied by Sinopharm Chemical Reagent Co., Ltd. (Shanghai, China). Lactate dehydrogenase (LDH) activity assay kit, 2,7-dichlorodihydrofluorescein diacetate (DCFH-DA) and crystal violet staining solution (0.4 wt%) were obtained from Solarbio Science &Technology Co., Ltd. (Beijing, China). Reduced cytochrome C (RCC) was procured from Abcam (Shanghai, China). Cell Counting Kit-8 (CCK-8) was supplied by Dojindo.

### 4.2 Characterization

The morphologies of the GSP NJs were characterized by TEM (FEI Tecnai G2 F20 U-TWIN) and scanning electron microscopy (SEM, Hitachi S-4800). The composition of the GSP NJs was measured by EDS and XPS (Thermo Fisher ESCALAB 250Xi). Raman spectra were recorded with an In-Via Renishaw spectrometer. The Ag^+^ ions release was determined by an inductively coupled plasma optical emission spectroscopy (ICP-OES, Thermo Scientific iCAP 6300). UV-VIS-NIR absorption spectra were collected by a multimode plate reader (PerkinElmer EnSpire). The zeta potential was measured using a Zetasizer (Malvern Zetasizer Nano ZS). Surface groups were analyzed by infrared spectroscopy (Perkin Elmer Spectrum One). Radical signals were detected by ESR (JEOL JES-FA200). Thermal images and temperature variations were measured using an infrared camera (Teledyne FLIR-6).

### 4.3 Synthesis of the GNBs

The GNBs were prepared using a seed-mediated growth method with slight modification (Sanchez-Iglesias et al., 2017). Briefly, a freshly prepared ice-cold NaBH_4_ solution (25 mM, 0.25 mL) was mixed with 10 mL of aqueous solution containing HAuCl_4_ (0.25 mM), citric acid (5 mM) and CTAC (50 mM) by vigorous stirring for 3 min. Then, the resulting mixture was heated at 85°C in an oil bath for 120 min, with a color change from brown to red, which was used as a seed solution. After cooling down to room temperature, 0.25 mL of the seed solution was injected into a growth solution premade by mixing CTAB (100 mM, 10 mL), HAuCl_4_ (10 mM, 0.5 mL), AgNO_3_ (10 mM, 0.1 mL), HCl (1 M, 0.2 mL) and AA (100 mM, 0.08 mL), followed by gentle inversion for 10 s. In the following, the above reaction solution was left undisturbed for 3 h at 30°C to allow the Au growth. Finally, the products were collected by centrifugation (8000 rpm, 10 min) and dispersed into 10 mL of water to obtain a GNB stock solution for further use. The as-prepared GNBs had length of ∼96 nm and width of ∼27 nm, with an aspect ratio (AR) of ∼3.5.

The above procedures were applied to synthesize other GNBs with different aspect ratios (2.6, 3.7, and 3.9), except that the volume of the seed solution (0.4, 0.15, and 0.08 mL) was changed.

### 4.4 Synthesis of the GS NRs

The GS NRs were synthesized by the overgrowth of Ag atoms on the GNBs. Briefly, to 5 mL of GNB stock solution in a scintillation vial, CTAC (160 mM, 5 mL), AgNO_3_ (10 mM, 0.6 mL) and AA (100 mM, 0.3 mL) were successively added. Then, the vial was placed in an air-bath shaker for 4.5 h (55°C, 120 rpm) to allow the Ag growth. Finally, the products were collected by centrifugation (5000 rpm, 10 min) and dispersed into 5 mL of water to obtain a GS NRs stock solution for further use. The as-prepared GS NRs had length of ∼145 nm and width of ∼38 nm, with an AR of ∼3.8.

To trace the morphological change of the GS NRs during the synthesis, the above procedures were applied, in which the various volume of the AgNO_3_ solution (100, 200, 350, 600, 900 and 1200 μL) and AA solution (50, 100, 175, 300, 450, 600 μL) were introduced.

### 4.5 Synthesis of the GSP NJs

The GSP NJs were obtained by etching the Ag shells of the GS NRs for the growth of *p*B. Take the synthesis of the GSP NJs with a PB thickness of 35 nm, for example, an aqueous etchant solution was first prepared by mixing K_3_ [Fe(CN)_6_] (10 mM, 0.6 mL) and FeNO_3_ (10 mM, 0.8 mL) with 3.6 mL of water. Then, to 2 mL of water in a round-bottomed flask, 0.35 mL of PVP solution (5 mg/mL), 1 mL of etchant solution and 1 mL of GS NR solution were added in sequence, followed by vigorous stirring for 30 min at room temperature. Finally, the products were collected by centrifugation (6000 rpm, 10 min) and washed with ethanol.

The GSP NJs with different thicknesses of the PB shells (6, 12, 21, 35, 45, 52 nm) were obtained by adjusting the feeding of AgNO_3_ (100, 200, 350, 600, 900 and 1200 μL), K_3_ [Fe(CN)_6_] (100, 200, 350, 600, 900 and 1200 μL) and FeNO_3_ (133, 267, 467, 800, 1200, 1600 μL), respectively.

To investigate the process of Ag etching and PB growth, different volume of the K_3_ [Fe(CN)_6_] solution (70, 150, 300 and 600 μL) and FeNO_3_ solution (93, 200, 400, 800 μL) were mixed to prepare the etchant solution. Besides, to study the effect of PVP on the dispersibility of the GSP NJs, a series of control experiments were performed by only changing the concentration of PVP (0, 1, 2, 5, 10 and 15 mg/mL).

### 4.6 Surface functionalization of the GSP NJs with vancomycin

Briefly, 0.5 mL of Van-HCl solution (500 μg/mL) was mixed with 5 mL of the GSP NJs (300 μg/mL). After gentle stirring overnight, the products were collected by centrifugation to remove excess vancomycin and redispersed in 5 mL of water.

### 4.7 SERS analysis

To study the bio-silent SERS properties of GSP NJs, the spectrum of the GSP NJs (300 μg/mL) was measured. For spectroscopic comparison, 300 μg/mL of GNBs was incubated with 10^−5^ M of RG6 and 4-ATP for 30 min, respectively, following by centrifugation and spectra measurement. To acquire the SERS spectra, a 785 nm Raman excitation light was selected and all the samples were tested in the liquid phase *via* a capillary tube, with the following parameters: a power of 50%, an accumulation time of 10 s and a 50x lens. The raw Raman data were further processed with baseline correction and signal smoothing to eliminate interference.

To compare the SERS responses of different materials in the bio-silent region, the spectra of the GSP NJs (300 μg/mL), GNBs (300 μg/mL) incubated with PB solution (300 μg/mL), PB powder, GNBs (300 μg/mL) and GS NRs (300 μg/mL) were collected.

### 4.8 Enzyme-mimic catalysis

To test the peroxidase-like activity, a solution containing the GSP NJs (10 μg/mL), TMB or ABTS (1 mM) and H_2_O_2_ (5 mM) in acetate buffer (pH = 3, 100 mM) was incubated for 5 min, followed by the UV-VIS measurement. To study the effects of temperatures and pH on the catalytic activities, the temperature was changed from 5 to 75°C and the pH was changed from 2 to 8, respectively, followed by absorbance measurement of oxidized TMB at 652 nm. The kinetic experiments were conducted based on our previous work (Cai et al., 2016).

### 4.9 Catalytic mechanism study

To test the possible hydroxyl radicals generated in the peroxidase-mimic catalysis by the GSP NJs, two types of probes (TA and DMPO) were used. In the TA experiment, TA (0.25 mM, 10 μL), H_2_O_2_ (50 mM, 10 μL), GSP NJ dispersion (300 μg/mL, 10 μL) were added in 470 μL of buffer (pH = 3) and incubated for 10 min, then the fluorescence spectrum of the above solution was collected. For comparison, a Fenton reaction was carried out using the same experimental parameters except the replacement of the GSP NJs by FeSO_4_ (50 mM, 10 μL). In the DMPO experiment, DMPO (500 mM, 10 μL), H_2_O_2_ (200 mM, 10 μL), GSP NJ dispersion (300 μg/mL, 10 μL) were added in 470 μL of buffer and incubated for 10 min, then the ESR spectrum of the above solution was collected. After that, a Fenton reaction as a reference was carried out using the same experimental parameters except the replacement of the GSP NJs by FeSO_4_ (200 mM, 10 μL).

To test the possible electron transfer, the oxidation of RCC was used as a probe reaction. Briefly, the RCC (5 mM, 10 μL) and GSP NJs (300 μg/mL, 20 μL) were incubated in buffer (pH = 3, 970 μL) under N_2_ atmosphere for 30 min, followed by the UV-VIS measurement.

### 4.10 Bacterial detection

The bacteria were cultured based on our previous work (Wen et al., 2022). For the detection, 1 mL of a mixed solution containing bacteria (0–10^8^ CFU/mL) and GSPv NJs (200 μg/mL) was prepared and incubated for 30 min in an air-bath shaker (37°C, 120 rpm), after which the bacteria-material complex was separated from the solution by centrifugation (2400 rpm, 5 min) and the collected precipitate was dispersed into 100 μL of water for the analysis of two modes. In the SERS mode, the samples were taken in capillary tubes and tested directly afterwards. In the nanozyme mode, 270 μL of buffer (pH = 3) was first heated at 55°C for 15 min. Then, TMB (30 mM, 10 μL), H_2_O_2_ (150 mM, 10 mL) and 10 μL of the resulting dispersion were added to the above buffer and incubated for 5 min, followed by the measurement of absorbance at 652 nm.

### 4.11 Photothermal activities

In a typical test, 500 μL of the GSP NJ dispersion (80 μg/mL) was irradiated with an 808 nm near-infrared (NIR) laser (0.8 W/cm^2^) for 10 min. Then, the temperature changes were recorded using an infrared camera every 30 s. For comparison, other materials (GNBs, GS NRs and GSPv NJs) were used for test under similar conditions.

To study the effect of concentration and laser power density on the photothermal heating, different concentrations of the GSP NJs (20, 40, 60, 80 and 100 μg/mL) and power density (0.4, 0.6, 0.8 and 1.0 W/cm^2^) were regulated, respectively. The photothermal stability of the GSP NJs was studied by repeating the heating/cooling processes for 5 cycles.

### 4.12 Photodynamic activity

The generation of reactive oxygen species (ROS) was probed by DPBF. Briefly, DPBF (20 μg/mL) and the materials (100 μg/mL) were mixed in 1 mL of water, followed by stirring in the dark for 2 h. Then, the above solution was irradiated by a NIR laser at a power density of 1 W/cm^-2^ for 10 min. Afterwards, the samples were taken out for UV-VIS measurements.

ESR was further used for the identification of ROS. To test possible ^1^O_2_, 10 μL of TEMP (2 M) and 125 μL of GSP NJs (400 μg/mL) were added into 365 μL of water, followed by incubation under irradiation for 10 min. To test possible •OH or O_2_^•−^ radicals, the above procedure was applied except that TEMP was replaced by DMPO (500 mM).

### 4.13 Ag^+^ release behavior

To determine the concentration of Ag^+^ ions in the solution released from the materials, 500 μL of the material dispersion (80 μg/mL) was taken, followed by NIR irradiation (0.8 W/mL). Subsequently, the treated samples were centrifuged to collect the supernatant for ICP analysis.

### 4.14 Antibacterial properties

The antibacterial experiments were carried out by a colony count method. To compare the antibacterial performance between different materials (GNBs, GS NRs, GSP NJs, and GSPv NJs), 10^6^ CFU/mL of bacteria were incubated with the materials (80 μg/mL) in 1 mL of water at 37°C for 1 h, followed by the treatments with or without NIR irradiation (0.8 W/cm^2^) for 5 min. Afterwards, the solution was diluted 25 times, and 50 μL of the diluted solution was dispersed onto Luria-Bertani plates for bacterial growth overnight. Then, the number of grown colonies was counted and the relative viability was calculated.

To study the concentration of the material on bacterial elimination, the GSPv NJs with different concentrations (20, 40, 60, 80, 100 μg/mL) were incubated with 10^6^ CFU/mL of bacteria, followed by the same procedures as above.

### 4.15 Cell viability assay

HUVEC cells were cultured in DMEM medium (with 15% FBS) in an incubator at 37 °C with a CO_2_ concentration of 5%. To study the cytotoxicity of the GSPv NJs, different concentrations of the NJs (0–200 μg/mL) were incubated with cells (10,000 cells/well) in the 96-well plates for 24 h. Cell viability was tested according to the standard procedure of the CCK-8 kit.

### 4.16 Antibacterial mechanism investigations

To characterize the morphological changes of bacteria, 10^7^ CFU/mL of bacteria were mixed with the GSPv NJs (100 μg/mL) in 500 μL of water with or without laser irradiation (0.8 W/cm^2^) for 10 min. After that, 10 μL of the mixed solution were dripped onto silicon wafers and fixed with 5% glutaraldehyde for 4 h. Then, the as-obtained samples were gradually dehydrated with a series of ethanol (30, 50, 70, 80, 90 and 100 wt%) for SEM analysis. The integrity of the bacterial membrane was determined by the standard procedures of a commercial LDH kit.

To study the oxidative damage of bacteria, a concentration of 10^8^ CFU/mL of bacteria were incubated with 10 μM of DCFH-DA at 37°C for 2 h, followed by the treatments of NIR irradiation (0.8 W/cm^2^, 10 min), GSPv NJs (80 μg/mL), and the combination (irradiation and GSPv NJs), respectively. After that, the fluorescence spectra of the above samples were collected, with an excitation and an emission wavelength of 488 nm and 525 nm, respectively.

### 4.17 Biofilm elimination

For biofilm degradation, a crystal violet staining method was adopted. Briefly, 500 μL of bacterial solution (10^8^ CFU/mL) was dispersed in trypsin broth (TSB) medium in the 24-well plate and then incubated for 48 h at 37 °C, in which TSB medium was replaced every 24 h. After incubation, different concentrations of the GSPv NJs (0, 50, 75 and 100 μg/mL) were added. The resulting mixtures were irradiated by NIR laser (1 W/cm^2^) for 20 min, followed by fixation in methanol for 30 min and staining with 0.2 wt% crystal violet for 15 min. Finally, the as-obtained samples were washed thoroughly with PBS for absorbance measurement.

### 4.18 Statistical analysis

All experiments are means of triplicates. The data were expressed as means ± standard deviation. The statistical significance was analyzed by using the Student’s t-test (for two groups only) and the one-way analysis of variance (ANOVA) with Turkey test (for groups more than two). The statistical significance was set at *p* < 0.05.

## Data Availability

The original contributions presented in the study are included in the article/Supplementary Material, further inquiries can be directed to the corresponding authors.
