# Supplementary material for: Versatile gold-silver-PB nanojujubes for multi-modal detection and photo-responsive elimination against bacteria
Source: Front Chem. 2023 May 22;11:1211523. doi: 10.3389/fchem.2023.1211523 (PMC10239827; doi:10.3389/fchem.2023.1211523)
Supplement: Supplementary file 1 [file DataSheet1.docx]

Supplementary Material

# Supplementary Theoretical simulation

An effective medium theory was used to calculate the absorption spectrum of GSP NJs with different thickness of shells (6, 12, 21, 35, 45, 52 nm) and illustrate the geometry dependence of the absorption rate. The GSP NJs were modeled as core-shell ellipsoids. The effective dielectric permittivity of the core-shell ellipsoid *ε_Au@S_* was calculated from the following formula:

$\varepsilon_{Au@S}=\varepsilon_{S}\frac{1-(1-L^{(s)})fG}{1+L^{(s)}fG}$ (1)

$G=\frac{\varepsilon_{S}-\varepsilon_{Au}}{L^{(c)}\varepsilon_{Au}+(1-L^{(c)})\varepsilon_{S}}$ (2)

where *L^(c)^*and *ε_Au_* are the geometric factor and dielectric permittivity of the Au core ellipsoid, *L^(s)^* and *ε_S_* are the geometric factor and dielectric permittivity of the shell, respectively. The absorption cross section *C_abs_* can be calculated by the formula:

$C_{abs}=k\cdot Im \left[ V_{Au@S}\frac{\varepsilon_{Au@S}-\varepsilon_{b}}{L^{(s)}\varepsilon_{Au@S}+(1-L^{(s)})\varepsilon_{b}} \right]$ (3)

where *k* is the wave vector, *V_Au@S_* is the volume of the NJs and *ε_b_* is the dielectric permittivity of the background with a value of 1.8. In the calculation, the finite size effect has been also taken into account (Kuwata et al., 2003). The geometric parameter *L* is related to the aspect ratio (AR) of the NJs, with a following relationship:

$L=\frac{1-e^{2}}{e^{2}}\left( \frac{1}{2e}\ln\left( \frac{1+e}{1-e} \right)-1 \right)$ (4)

$e=\sqrt{1-(1/AR)^{2}}$ (5)

Given the shape fluctuations of the NJs, we have performed the average over the geometric parameter *L* with Gaussian distribution of width 0.01. The dielectric permittivity of the shell (the mixture of PB and Ag) was obtained as follows: *ε_S_* = *qε_PB_* + (1-*q*) *ε_Ag_*, where the fitting parameter *q* is 0.95, *ε_Ag_* and *ε_Au_* were obtained from the reference (Johnson et al., 1972). The dielectric permittivity of PB was obtained based on the formula (Nesterov et al., 2016):

$\varepsilon_{PB}=\varepsilon_{b}-\sum_{i=1,2} A_{i}\left( \frac{1}{\hbar\omega-\hbar\omega_{i}+i\gamma_{i}}-\frac{1}{\hbar\omega+\hbar\omega_{i}+i\gamma_{i}} \right)$ (6)

To match the absorption spectrum of PB (Jiang et al., 2018), the parameters were chosen as follows: *A_i_* = 0.2/0.25*eV*, *ℏω_i_* = 4.28/1.66*eV*, *γ_i_* = 0.5/0.3*eV*, and *i* = 1, 2.

# Supplementary Figures and Tables

## Supplementary Figures


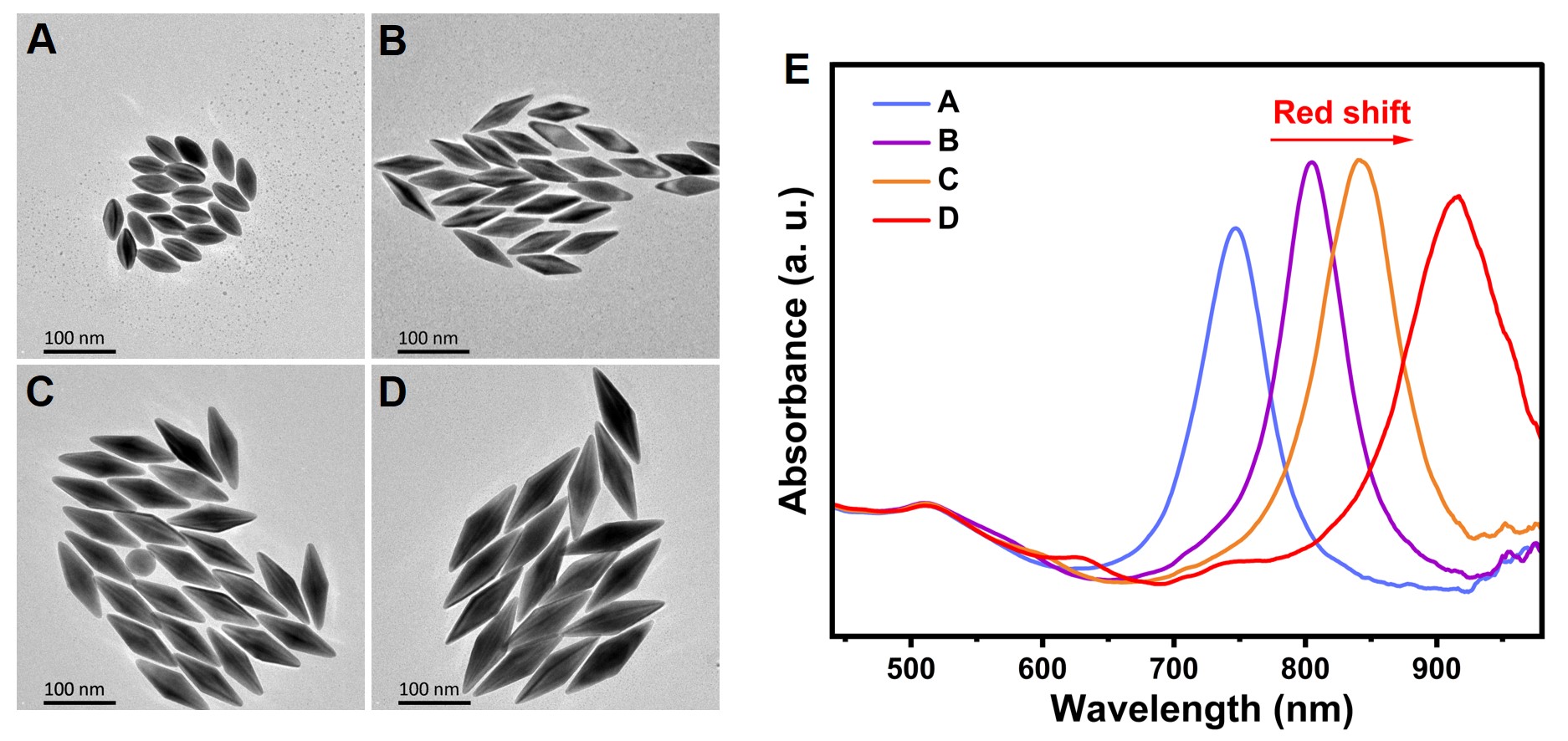


**Supplementary Figure S1.** Characterization of the GNBs. (A-D) TEM images of the GNBs with increasing AR (from 2.6 to 3.9) and (E) corresponding UV-VIS-NIR spectra of the GNBs.


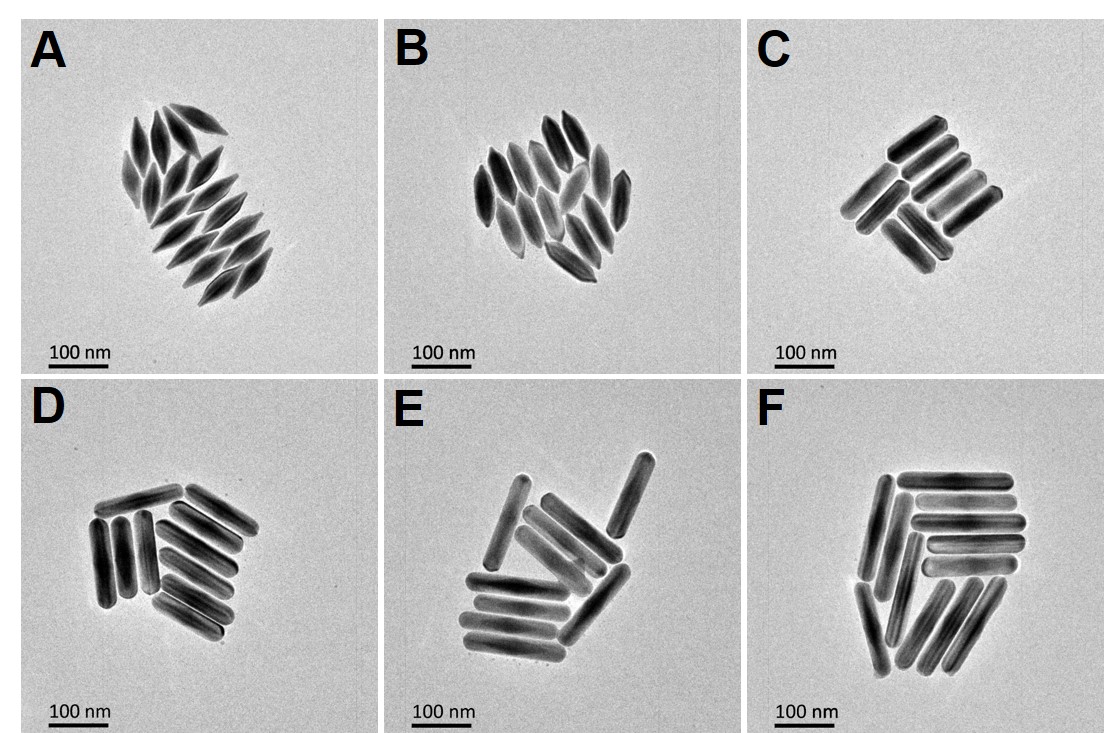


**Supplementary Figure S2.** Characterization of the GS NRs. (A-F) TEM images of the various GS NRs showing the effect of the AgNO_3_ usage on their morphologies.


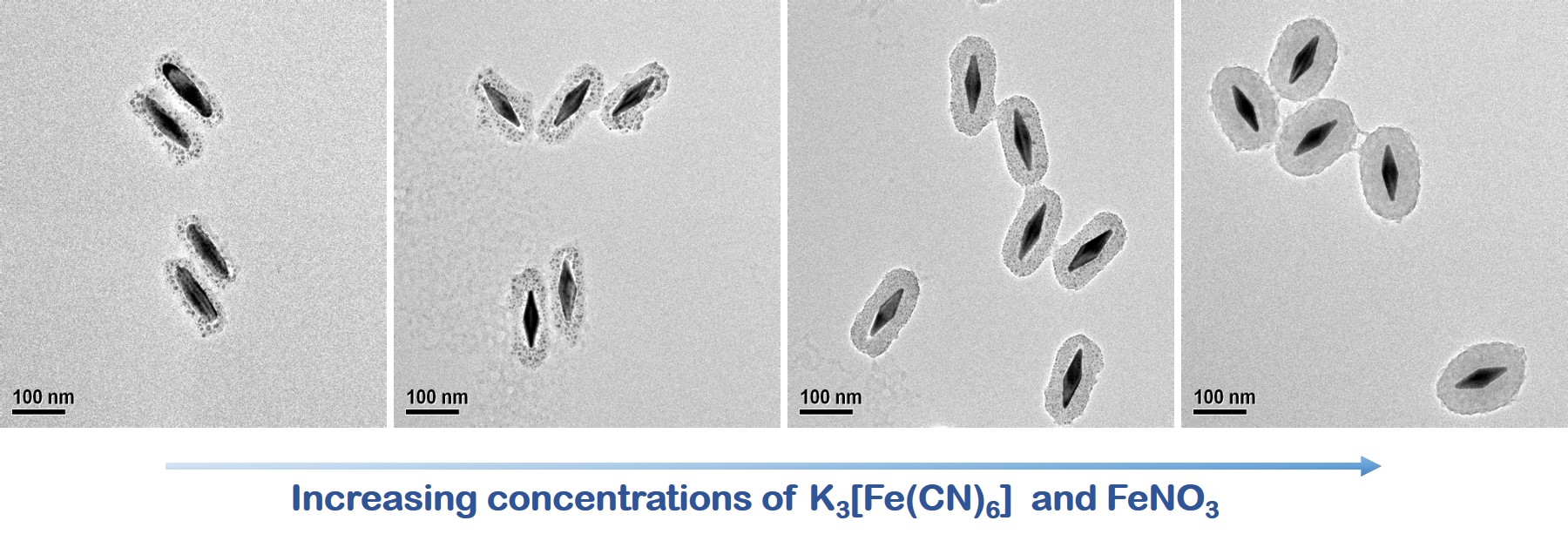


**Supplementary Figure S3.** TEM images of the intermediates showing the Ag etching and PB growth by raising the concentrations of K_3_[Fe(CN)_6_] and FeNO_3_ as the etchants.


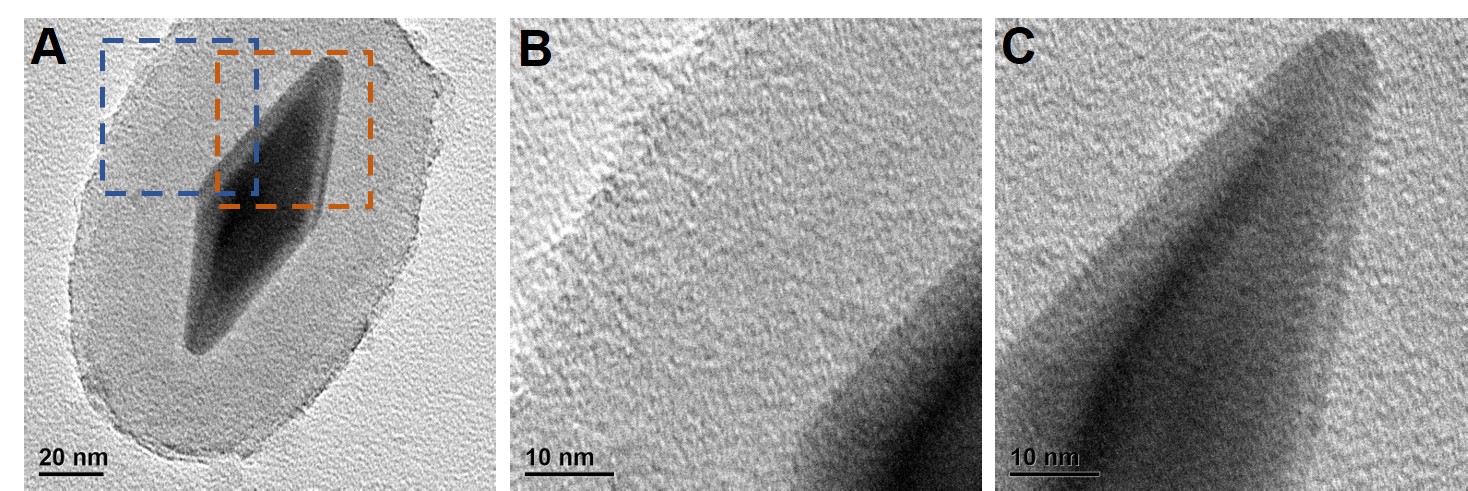


**Supplementary Figure S4.** High-resolution TEM images of the GSP NJs. (A) TEM image of a single GSP NJ. (B) High-resolution TEM image of the blue region. (C) High-resolution TEM image of the orange region. There were no Ag nanoparticles in large size in the PB shell layer.


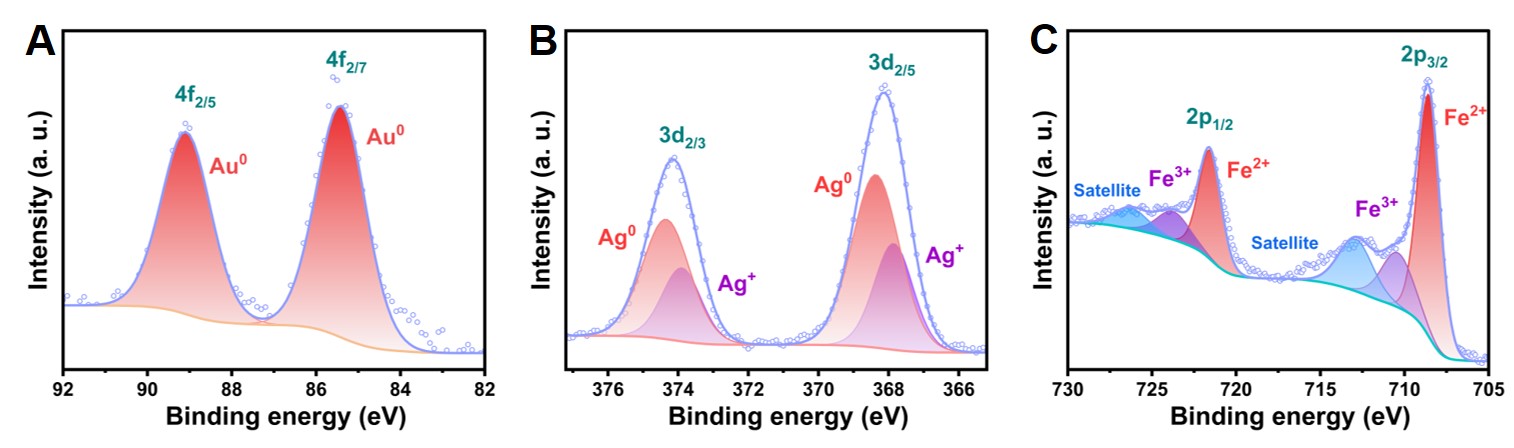


**Supplementary Figure S5.** High-resolution XPS spectra of (A) Au 4f, (B) Ag 3d and (C) Fe 2p regions of the GSP NJs.


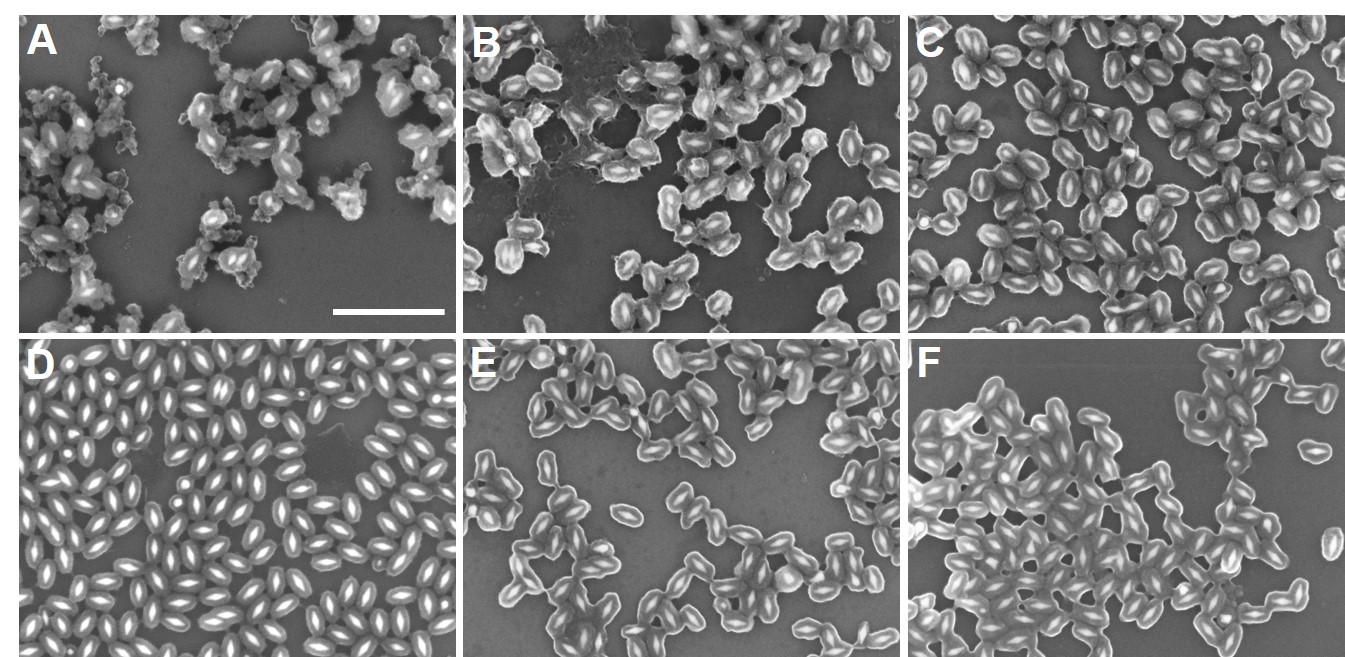


**Supplementary Figure S6.** Characterization of the GSP NJs obtained by different usage of PVP as a stabilizer. (A-F) SEM images of the GSP NJs showing the effect of the PVP concentration (0, 1, 2, 5, 10 and 15 mg/mL) on the dispersibility of the GSP NJs. Scale bar: 500 nm.


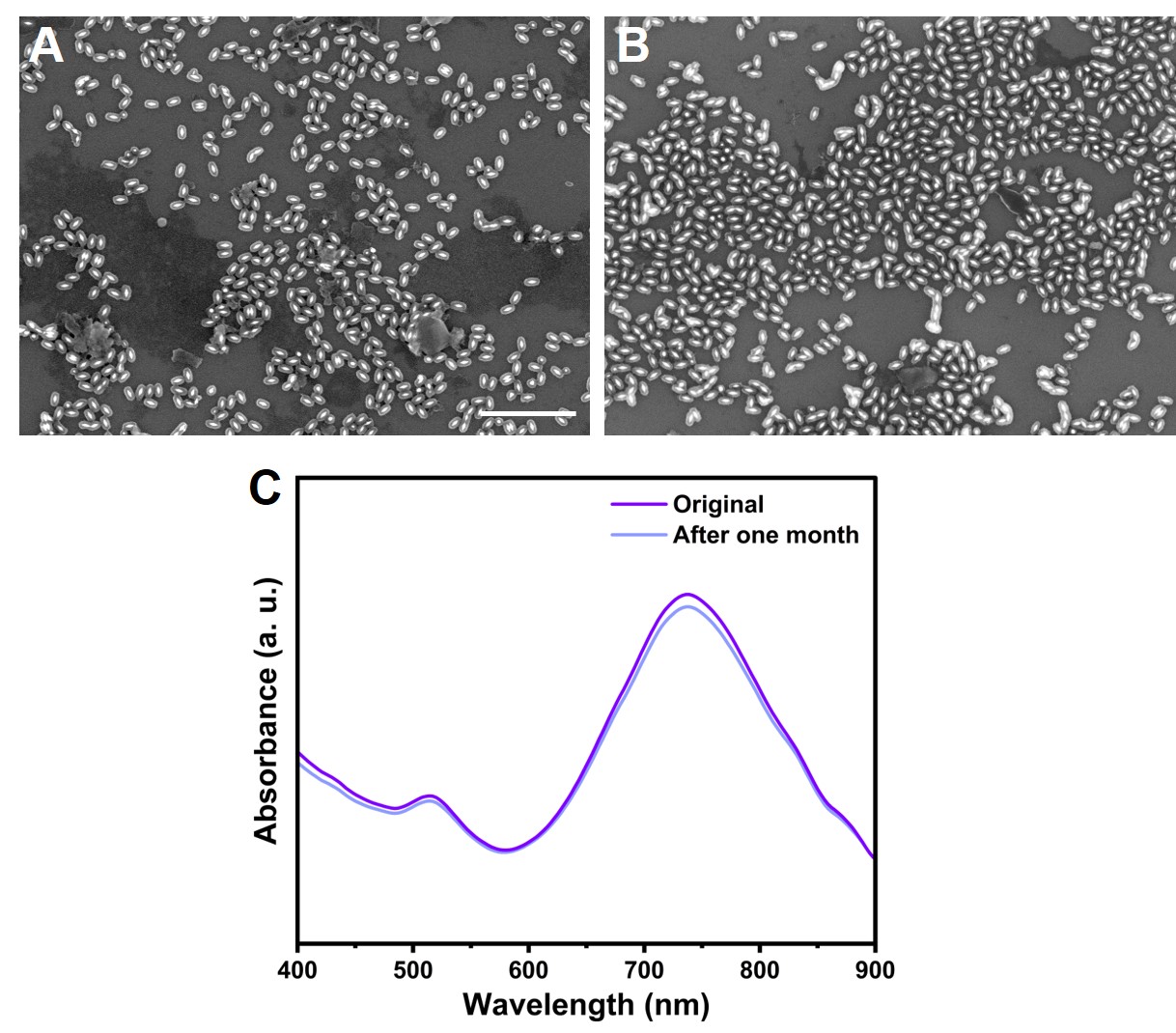


**Supplementary Figure S7.** The storage stability of the GSP NJs. SEM images of (A) original sample and (B) sample after one month of storage. Scale bar: 1 μm. (C) The corresponding UV-VIS-NIR spectrum.


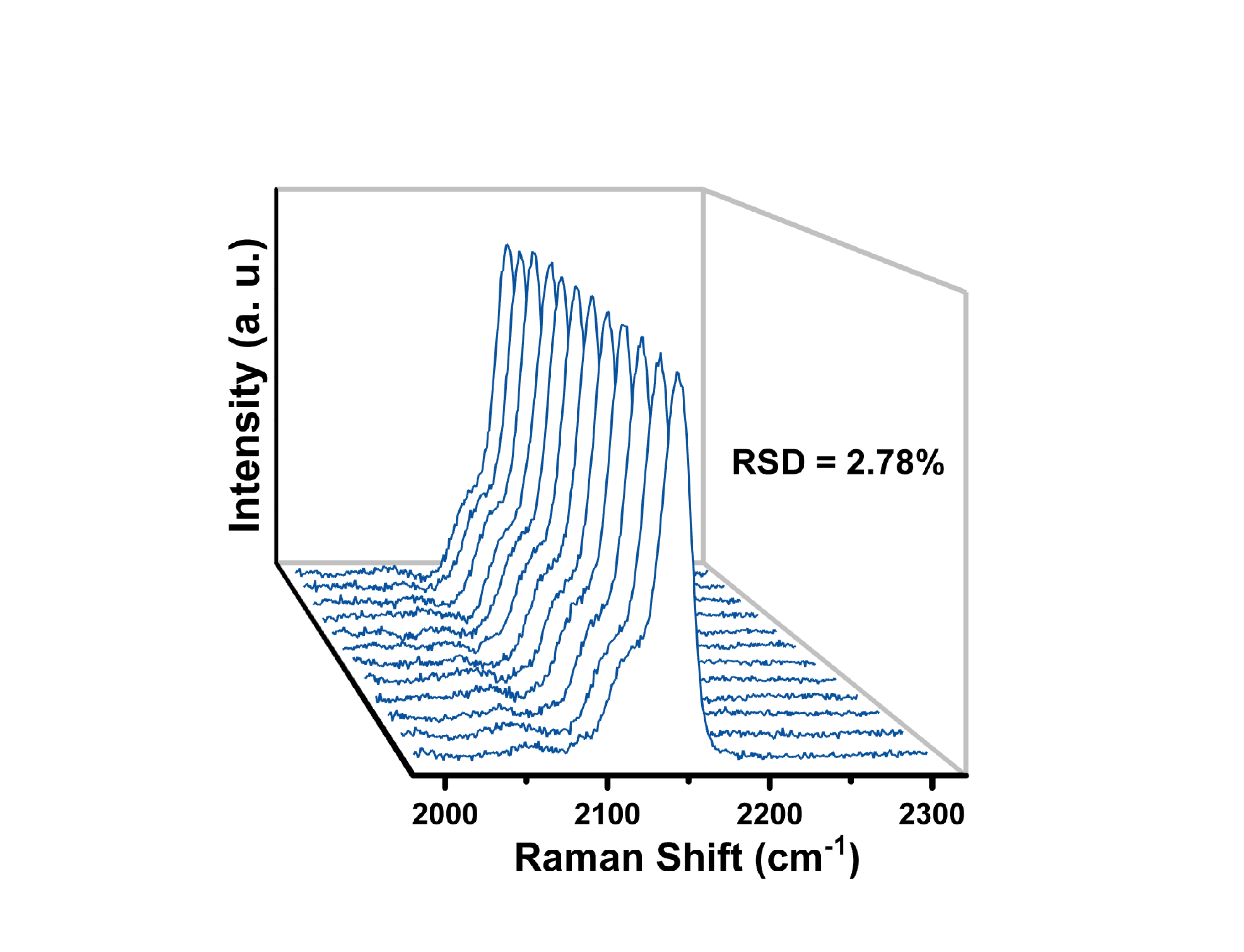


**Supplementary Figure S8.** The reproducibility of the SERS signal obtained by the GSP NJ samples taken at 12 locations randomly selected.


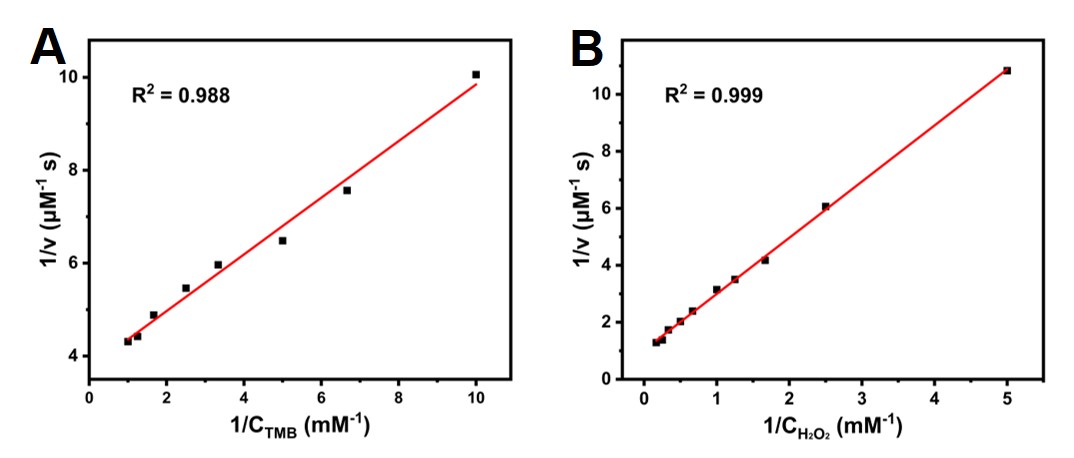


**Supplementary Figure S9.** Catalytic kinetics of the GSP NJs analyzed by Lineweaver-Burk model toward the substrate of (A) TMB and (B) H_2_O_2_.


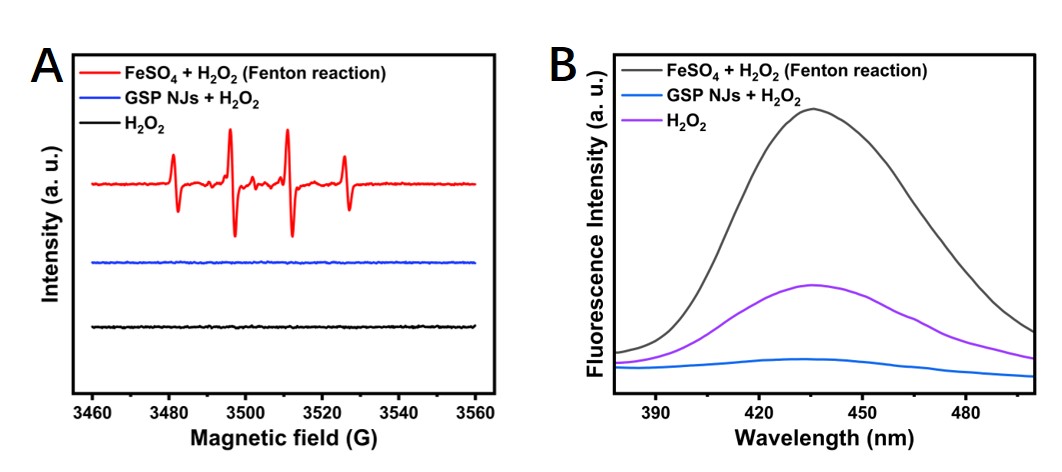


**Supplementary Figure S10.** Spectroscopic investigations on the generation of hydroxyl radicals during the peroxidase-like catalysis of the GSP NJs. The standard Fenton reaction was used as the reference. (A) ESR spectra collected with DMPO as a trap for different reaction systems. (B) Fluorescence spectra collected with TA as a probe for different reaction systems.





**Supplementary Figure S11.** UV-VIS-NIR spectra of different reaction solutions for the oxidations of the RCC showing electron transfer.





**Supplementary Figure S12.** High-resolution XPS spectrum of Au 4f region of the GNBs.


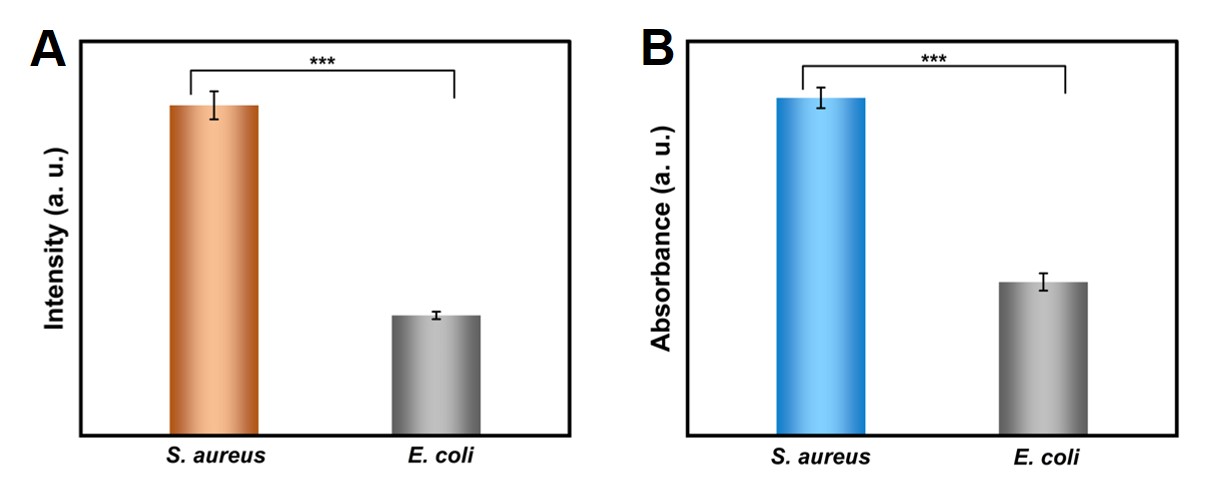


**Supplementary Figure S13.** Selectivity of the GSPv NJs for bacterial detection. The signal intensity was compared between different bacteria at a concentration of 10^7^ CFU/mL in (A) SERS mode and (B) nanozyme mode. Error bars indicate means ± standard deviations (*n* = 3 biologically independent samples). Statistical significance was analyzed by Student’s test: *P < 0.05, **P < 0.01 and ***P < 0.001.


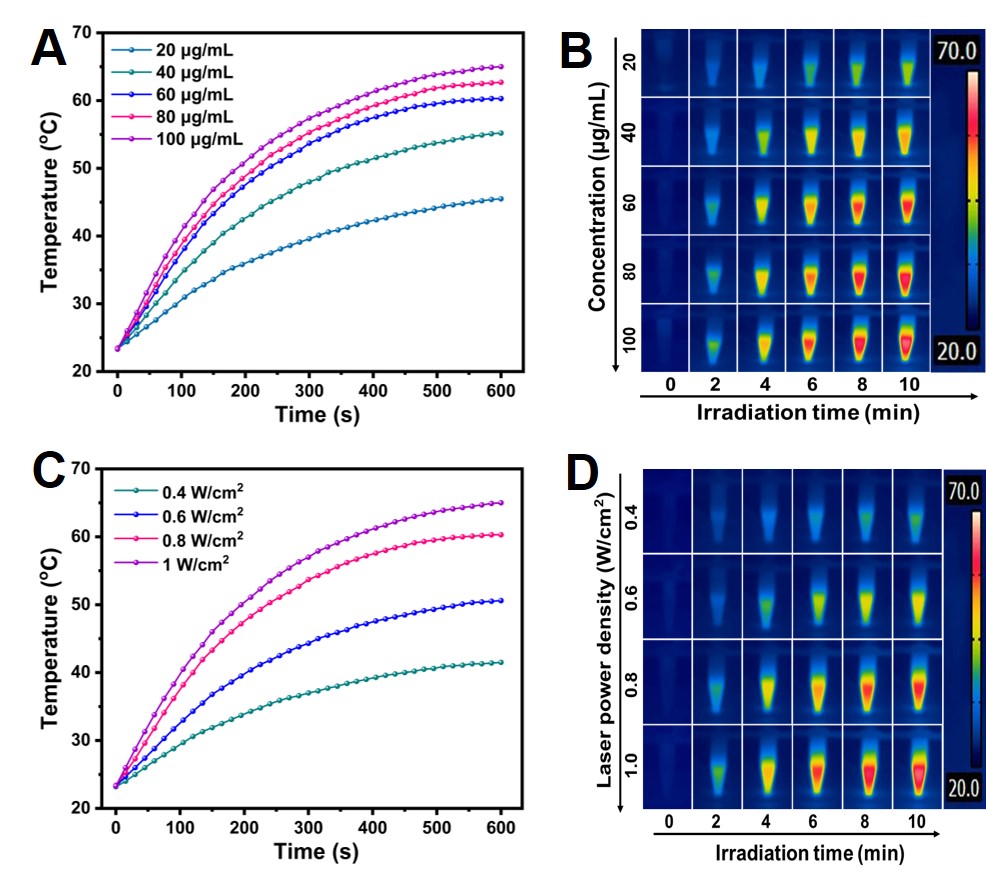


**Supplementary Figure S14.** PTT activity of the GSP NJs. (A) Effect of the concentration on the PTT heating curves and (B) the corresponding real-time infrared thermal images. (C) Effect of the laser power density on the PTT heating curves and (D) the corresponding real-time infrared thermal images.


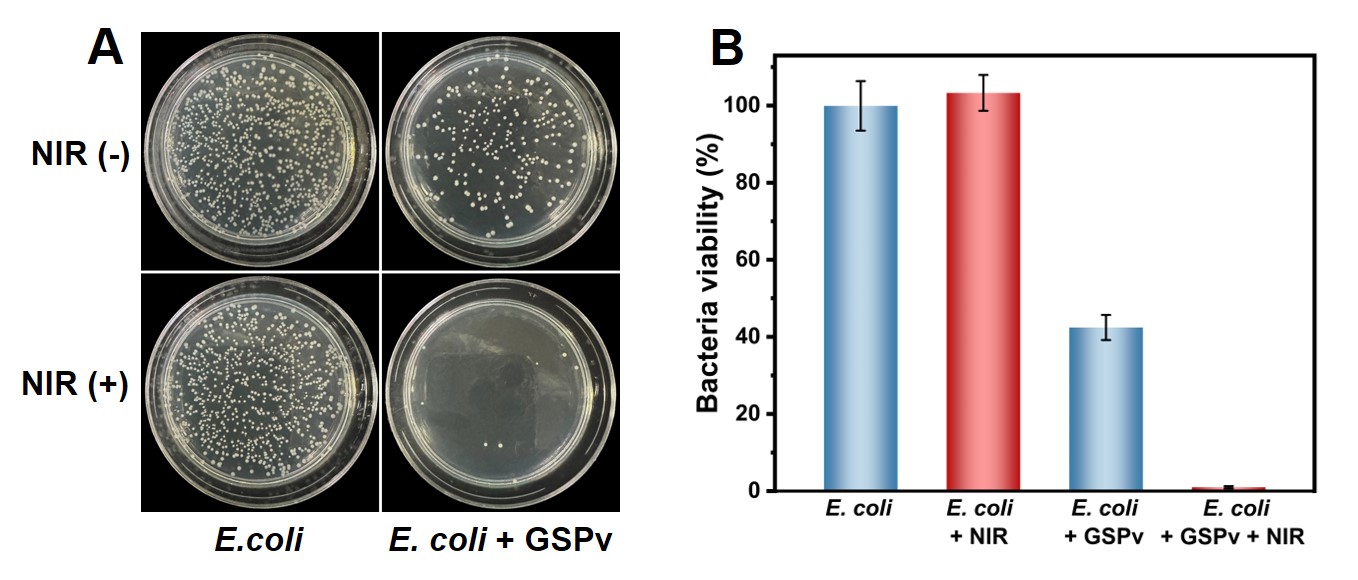


**Supplementary Figure S15.** Antibacterial performance of the GSPv NJs. (A) Photographs of *E. coli*. colonies incubated on agar plates under different treatment of the GSPv NJs and NIR irradiation. (B) The corresponding statistics of colonies on the plates.





**Supplementary Figure S16.** Cytotoxicity of the GSPv NJs after incubating with cells for 24 h analyzed by the CCK-8 assay.

## Supplementary Tables

**Supplementary Table S1.** Catalytic parameters of different catalysts.

| **Catalyst** | **Substrate** | ***k*_m_ (mM)** | ***v*_max_ (10^-8^ M·s^-1^)** |
| --- | --- | --- | --- |
| **GSP NJs** | **TMB** | **0.161** | **26.6** |
|  | **H_2_O_2_** | **2.33** | **108** |
| **HRP**  **(Gao et al., 2007)** | **TMB** | **0.434** | **10** |
|  | **H_2_O_2_** | **3.7** | **8.71** |

**Supplementary Table S2.** Comparison of *S. aureus* detection between different methods.

| **Methods** | **LOD (CFU/mL)** | **Assay duration** | **Reference** |
| --- | --- | --- | --- |
| **PCR** | **300** | **4 h** | **Yang et al., 2019** |
| **ELISA** | **4 × 10^3^** | **1.5 h** | **Yu et al., 2016** |
| **Fluorescence** | **33** | **2 h** | **Meng et al., 2017** |
| **Electrochemiluminescent** | **3 × 10^2^** | **70 min** | **Yue et al., 2016** |
| **Piezoelectric** | **41** | **60 min** | **Lian et al., 2015** |
| **Colorimetric** | **6.4 × 10^2^** | **50 min** | **Zhou et al., 2023** |
| **SERS** | **56** | **40 min** | **This work** |
| **Nanozyme** | **6** | **60 min** | **This work** |

# References

Gao, L. Z., Zhuang, J., Nie, L., Zhang, J. B., Zhang, Y., Gu, N., Wang, T. H., Feng, J., Yang, D. L., Perrett, S., and Yan, X. (2007). Intrinsic peroxidase-like activity of ferromagnetic nanoparticles. *Nat. Nanotechnol.* 2 (9)**,** 577-583. doi:10.1038/nnano.2007.260

Jiang, T. T., He, J. J., Sun, L., Wang, Y. L., Li, Z. L., Wang, Q., Sun, Y., Wang, W., and Yu, M. (2018). Highly efficient photothermal sterilization of water mediated by Prussian blue nanocages. *Environ. Sci.: Nano* 5 (5)**,** 1161-1168. doi:10.1039/c7en01245d

Johnson, P. B., and Christy, R. W. (1972). Optical Constants of the Noble Metals. *Phys. Rev. B* 6 (12)**,** 4370-4379. doi:10.1103/PhysRevB.6.4370

Kuwata, H., Tamaru, H., Esumi, K., and Miyano, K. (2003). Resonant light scattering from metal nanoparticles: Practical analysis beyond Rayleigh approximation. *Appl. Phys. Lett.* 83 (22)**,** 4625-4627. doi:10.1063/1.1630351

Lian, Y., He, F., Wang, H., and Tong, F. (2015). A new aptamer/graphene interdigitated gold electrode piezoelectric sensor for rapid and specific detection of *Staphylococcus aureus*. *Biosens. Bioelectron.* 65**,** 314-319. doi:10.1016/j.bios.2014.10.017

Meng, X., Yang, G., Li, F., Liang, T., Lai, W., and Xu, H. (2017). Sensitive detection of *Staphylococcus aureus* with vancomycin-conjugated magnetic beads as enrichment carriers combined with flow cytometry. *ACS Appl. Mater. Interfaces* 9 (25)**,** 21464-21472. doi:10.1021/acsami.7b05479

Nesterov, M. L., Yin, X. H., Schaferling, M., Giessen, H., and Weiss, T. (2016). The role of plasmon-generated near fields for enhanced circular dichroism spectroscopy. *Acs Photonics* 3 (4)**,** 578-583. doi:10.1021/acsphotonics.5b00637

Yang, G. T., Huang, M., Wang, Y. T., Chen, G. H., Zhao, Y., and Xu, H. Y. (2019). Streptavidin-exposed magnetic nanoparticles for lectin magnetic separation (LMS) of *Staphylococcus aureus* prior to three quantification strategies. *Microchim. Acta* 186 (12), 813. doi:10.1007/s00604-019-3978-4

Yu, J., Zhang, Y., Zhang, Y., Li, H., Yang, H., and Wei, H. (2016). Sensitive and rapid detection of *Staphylococcus aureus* in milk via cell binding domain of lysin. *Biosens. Bioelectron.* 77**,** 366-371. doi:10.1016/j.bios.2015.09.058

Yue, H., Zhou, Y., Wang, P., Wang, X., Wang, Z., Wang, L., and Fu, Z. (2016). A facile label-free electrochemiluminescent biosensor for specific detection of *Staphylococcus aureus* utilizing the binding between immunoglobulin G and protein A. *Talanta* 153**,** 401-406, doi:10.1016/j.talanta.2016.03.043

Zhou, B., Ye, Q., Chen, M., Wang, C., Xiang, X., Li, Y., Zhang, J., Zhang, Y., Wang, J., Wu, S., Gu, Q., Ding, Y., and Wu, Q. (2023). A label-free AuNP bioprobe-assisted CRISPR/Cas12a colorimetric platform for high-throughput detection of *Staphylococcus aureus* ST398. *Food Control* 145,109451. doi:10.1016/j.foodcont.2022.109451
